# A spam detection model based on the discriminative TF-IDF belief rule base

**DOI:** 10.1038/s41598-026-42223-6

**Published:** 2026-03-04

**Authors:** Xiting Yang, Wenkai Zhou, Xiping Duan, Ning Ma, Yuhe Wang

**Affiliations:** School of Computer Science and Information Engineering, Harbin Normal University, Harbin, 150025 China

**Keywords:** Computational biology and bioinformatics, Mathematics and computing

## Abstract

Novel spam with rapidly evolving content faces a scarcity of labeled data in its early stages. Yet, current detection models rely heavily on large datasets and high-dimensional features, leading to poor generalization and opaque decisions when data is scarce. This opacity hinders error tracing and limits their use in early threat detection and response. The belief rule base (BRB), as an expert system, demonstrates effective learning under small-sample conditions, and its rule-based reasoning mechanism provides decision interpretability. However, high-dimensional features may cause combination explosion. To address these issues, a BRB spam detection model based on the Discriminative term frequency-inverse document frequency (TF-IDF) method (DTI-BRB) is proposed in this paper. By discriminating whether terms are more indicative of ham or spam, the Discriminative TF-IDF method converts raw text into low-dimensional features, thereby effectively resolving the combination explosion problem inherent in the traditional BRB model. Through two case studies under small-sample conditions, the effectiveness of the proposed model is validated. With only 200 samples, it achieves accuracies of 91.5% and 95.5% in the two cases, respectively, exhibiting excellent predictive performance and interpretability.

## Introduction

The widespread use of email has made it a primary channel for spam propagation. Numerous cybersecurity research reports indicate that spam accounts for a persistently high proportion of global email traffic, causing ongoing disruption to both corporate and individual users^1,2^. This not only poses significant threats to user information security and privacy protection^3,4^, but also substantially consumes network bandwidth and storage resources. However, during the rapid response to novel spam in its initial outbreak phase, particularly types that evade detection primarily through textual variation, labeled samples available for model training are often limited^5^. With scarce samples, an interpretable and trustworthy decision-making process becomes crucial. Therefore, building a spam detection system capable of learning under small-sample conditions while providing a transparent decision process is of great importance^6^.

Since the mid-to-late 1990 s, researchers have proposed various methods for spam detection. These methods can be broadly categorized into three types: traditional machine learning models, deep learning models, and fuzzy systems. Traditional machine learning models rely on feature engineering to uncover statistical patterns from structured data. For example, Dedeturk et al. proposed a spam detection method combining an artificial bee colony algorithm with a logistic regression (LR) classifier. The method offers efficient local and global search capabilities for handling high-dimensional data^7^. Faris et al. developed a feature extraction tool for email corpora, with which they evaluated four models: multilayer perceptron (MLP), naive bayes (NB), random forest (RF), and decision tree (DT)^8^. Olatunji et al. compared support vector machine (SVM) and extreme learning machine (ELM) for email spam detection, with SVM achieving higher accuracy while ELM demonstrated significantly faster runtime^9^. Zheng et al. proposed a supervised ELM-based method that detects spammers by extracting features from message content and user behavior on a labeled dataset^10^. Rayan introduced a hybrid bagging method that combines RF and J48, whereby the dataset is divided into subsets fed into each method for spam detection^11^. Although these traditional machine learning-based methods achieve automated classification to some extent, their performance heavily depends on the dimension of features.

Deep learning models employ deep neural network architectures to achieve end-to-end feature learning, enabling automatic capture of multi-level abstract representations in data. For instance, Zavrak et al. proposed a spam detection framework integrating convolutional neural network (CNN), gated recurrent unit (GRU), and attention mechanisms. In this architecture, convolutional layers extract hierarchical features, while temporal convolutions enhance attention-based methods to achieve more abstract and generalizable representations^12^. Nasreen et al. enhanced feature selection from the original dataset by integrating deep learning techniques like CNN, bidirectional long short-term memory (BiLSTM), and long short-term memory (LSTM), achieving notable results^13^. Giri et al. combined binary unique number of word (BUNOW) and global vectors for word representation (GloVe) embeddings with deep learning models, creating an approach applicable to tasks such as spam detection, sentiment analysis, and text classification^14^. Doshi et al. proposed a two-layer architecture incorporating artificial neural networks (ANN), recurrent neural networks (RNN), and CNN to effectively address data imbalance in email phishing and spam classification^15^. Although deep learning models deliver strong performance, their training process consumes substantial computational resources and involves complex hyperparameter tuning.

Fuzzy systems are founded on fuzzy set theory and handle uncertainty by simulating human approximate reasoning mechanisms. For example, Atacak et al. utilized four interval type-1/type-2 fuzzy systems, finding that these fuzzy logic models outperformed machine learning alternatives in accuracy, recall, F1-score^16^. Khan et al. proposed a novel fuzzy logic-based metric, mu(O), integrating accuracy, recall, and precision, with a proof-of-concept using bidirectional encoder representations from transformers (BERT) and LSTM validating its effectiveness^17^. Juneja et al. proposed a hybrid spam model with a two-stage fuzzy feature filter, which identifies key features and integrates NB with RF via majority voting for probability-based detection^18^. Wang et al. proposed a fast content-based spam filtering algorithm using fuzzy support vector machines (FSVM) and k-means, effectively improving both the speed and accuracy of spam filtering^19^. Though fuzzy system-based methods demonstrate significant potential in providing decision transparency, they are prone to the curse of dimensionality when processing sparse features, leading to a notable decline in reasoning efficiency.

Although the aforementioned methods can be applied to spam detection, two critical challenges remain unaddressed. First is the problem of learning from small samples. Existing models rely on large datasets to ensure performance. During the initial outbreak of novel spam, when available samples are scarce, these models struggle to adapt quickly and maintain stable recognition capabilities. Second, the need for interpretability in model decisions. Most current methods exhibit opaque reasoning logic during classification, which not only undermines trust in the models’ outputs, but also complicates system optimization and error diagnosis.

To address these challenges, the belief rule base (BRB) is introduced in this paper. Proposed by Yang et al.^20^, the BRB is a typical expert system model capable of effectively integrating qualitative expert knowledge with quantitative data in the form of IF-THEN rules. The initialization of the BRB model incorporates expert knowledge into three key components: the reference points of the input attributions, the weights assigned to each rule, and the belief degrees within each rule’s consequent. This structured approach ensures that domain expertise directly shapes the model’s initial inference framework before data-driven optimization. The BRB can handle uncertainty in reasoning and maintain complete transparency in the decision-making process. Moreover, due to its dual advantages of being knowledge-driven and capable of uncertainty fusion, the BRB is particularly suitable for small-sample conditions. Currently, the BRB models have been applied in various fields such as fault detection^21–23^, health assessment^24–26^, and educational evaluation^27,28^. However, the application of the BRB models in text classification remains in its early stages. A comprehensive review of the relevant literature revealed no prior studies that specifically adapted the BRB for spam detection.

In the traditional BRB models, using high-dimensional text features of spam as input attributes would trigger the problem of combination explosion, making it impossible to address spam detection. To overcome this problem, in this paper, a BRB spam detection model based on the Discriminative term frequency-inverse document frequency (TF-IDF) method (DTI-BRB) is proposed. First, feature dimensionality reduction is performed using the Discriminative TF-IDF method, with the reduced-dimensional features serving as the input attributes of the model. Then, evidential reasoning rule is adopted as the inference mechanism. Finally, the projected covariance matrix adaptation evolution strategy (P-CMA-ES) algorithm is applied to optimize the initial parameters. Two case studies show that the model reduces sample requirements while maintaining excellent detection performance and interpretability, providing a new solution for reliable spam detection in small-sample conditions.

The main contributions of this paper are as follows:

1. This paper introduces the BRB to the field of spam detection for the first time and proposes the DTI-BRB spam detection model.
2. Through five-fold cross-validation and comparative experiments on multiple small-sample datasets, it is verified that the DTI-BRB model can effectively handle the condition of scarce samples during the initial outbreak of novel spam, while maintaining good applicability and interpretability.
3. The Discriminative TF-IDF method is proposed as an enhancement to the traditional TF-IDF method, which effectively solves the combination explosion problem. An ablation experiment removing the discriminative mechanism further demonstrates that this method can reduce feature dimensionality while preserving excellent model performance.

## Problem formulation

When developing the spam detection model, the following problems must be considered to ensure feasibility and reliability:

Problem 1: The method for feature dimensionality reduction. After preprocessing, raw text data is represented as high-dimensional feature vectors. Using these features would generate excessive rules, increasing model complexity. Therefore, the original features necessitate dimensionality reduction. This process can be described as follows:

1 $$\begin{aligned} X' = F(X) \end{aligned}$$

where *X* denotes the original feature set. $F(\cdot )$ denotes the mapping function. $X'$ denotes the transformed feature set.

Problem 2: The inference process for mitigating overfitting risk in small-sample conditions. With limited samples, the model should maintain robust inference capability. This process can be described as follows:

2 $$\begin{aligned} y = G(X', \Phi ) \end{aligned}$$

where $G(\cdot )$ denotes the inference function. $\Phi$ denotes the parameter set. *y* denotes the output result.

Problem 3: The optimization algorithm for mitigating the impact of expert knowledge limitations. Initial BRB parameters rely on expert knowledge. Therefore, effective optimization is essential. This process can be described as follows:

3 $$\begin{aligned} \mathrm {\Phi }_{\textrm{best}}=O\left( {\Phi }\right) \end{aligned}$$

where $\Phi _{\textrm{best}}$ denotes the optimized parameter set. $O(\cdot )$ denotes the optimization algorithm.

## DTI-BRB spam detection model

This section begins by establishing the basic structure of the DTI-BRB model, which is organized into three core components: the Discriminative TF-IDF feature extraction, the inference process, and the optimization process. Each is then detailed before providing a final summary of the modeling process.

### Basic structure of the DTI-BRB model

The BRB models consist of a series of belief rules, where the $k_{th}$ rule can be defined as follows:

4 $$\begin{aligned} \begin{aligned} R_k:&\ \text {IF } x_1 \text { is } A_1^k \wedge x_2 \text { is } A_2^k \wedge \cdots \wedge x_M \text { is } A_M^k, \\&\ \text {THEN } \{(D_1, \beta _{1,k}), (D_2, \beta _{2,k}), \ldots , (D_N, \beta _{N,k})\} \\&\ \text {with rule weight } \theta _k \text { and attribute weights } \delta _1, \delta _2, \ldots , \delta _M \end{aligned} \end{aligned}$$

where $x_i\left( i=1,2,\ldots ,M\right)$ denotes the $i_{th}$ input attribute of the model. $A_i^k$ denotes the reference value of the $i_{th}$ input attribute in the $k_{th}$ rule. $D_n\left( n=1,2,\ldots ,N\right)$ denotes the $n_{th}$ evaluation grade. $\beta _{n,k}$ denotes the belief degree associated with evaluation grade $D_n$ in the $k_{th}$ rule, satisfying $\sum _{n=1}^{N}\beta _{n,k}=1$. $\theta _k$ denotes the rule weight of the $k_{th}$ rule. $\delta _i$ denotes the attribute weight of the $i_{th}$ input attribute.

**Fig. 1:**
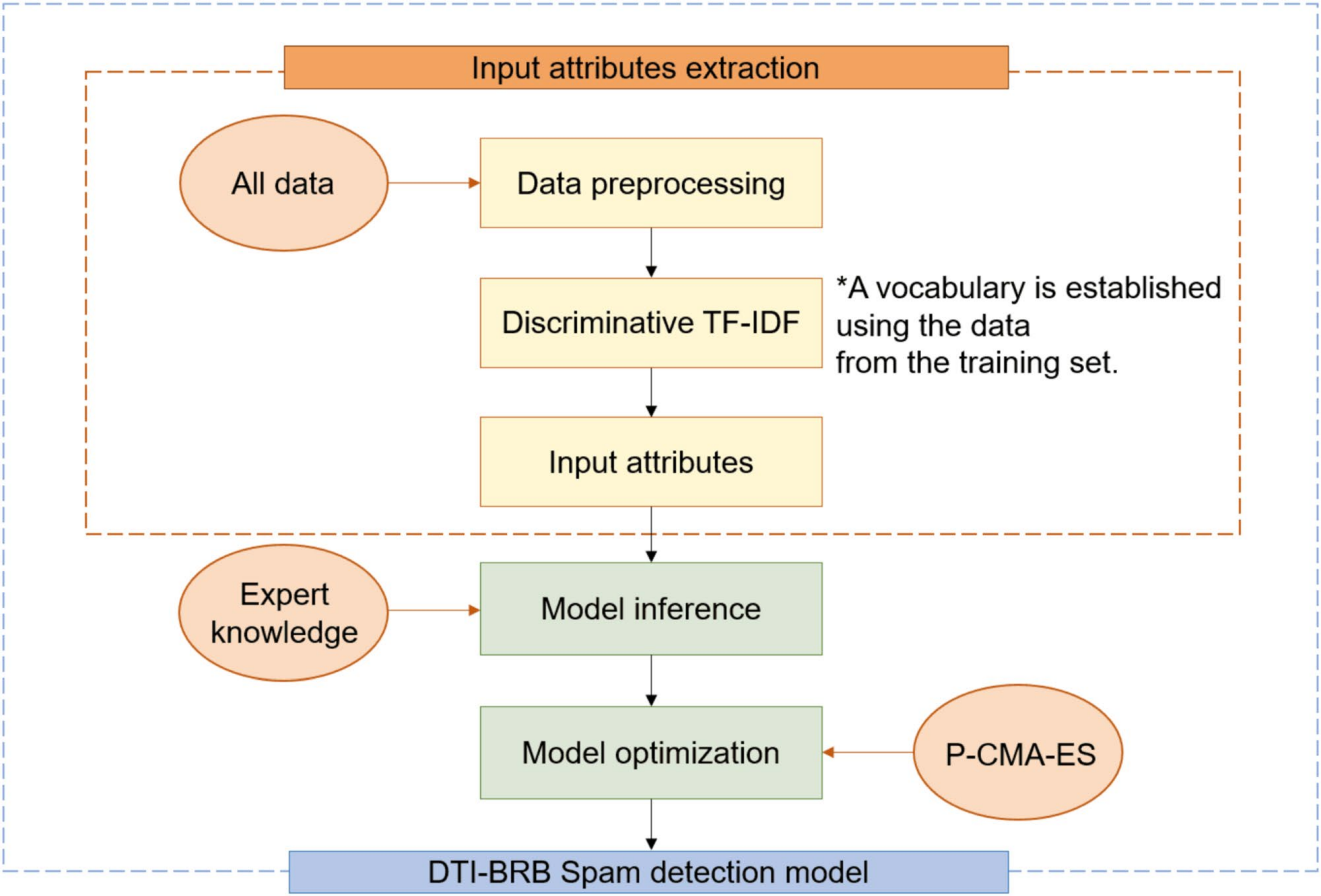
Structure of the DTI-BRB model.

Based on the rules, the core of the DTI-BRB model is the Discriminative TF-IDF method. Obtain input attributes with this method, then conduct model inference, and finally optimize the model. The basic structure of the DTI-BRB model is illustrated in Fig. 1.

### The discriminative TF-IDF method

The TF-IDF method performs feature extraction by evaluating the importance of terms in documents^29^. Term frequency (TF) measures how frequently a term appears in a document, and is defined as follows:

5 $$\begin{aligned} tf(t,d)=\frac{f_{t,d}}{\displaystyle \sum _{t' \in d} f_{t',d}} \end{aligned}$$

where *t* denotes a specific term. *d* denotes a specific document. $f_{t,d}$ denotes the frequency of term *t* in document *d*. $t'$ denotes a summation variable that iterates over every term in *d*.

Inverse document frequency (IDF) reflects how rare a word is across the entire corpus, and is defined as follows:

6 $$\begin{aligned} idf\left( t,D\right) =log\frac{Z}{1+\left| \{d\in D:t\in d\}\right| } \end{aligned}$$

where *D* denotes the document collection. *Z* denotes the total number of documents in the document collection *D*. $\left| {d\in D:t\in d}\right|$ denotes the number of documents containing the *t*.

The TF-IDF value of *t* in *d* is then defined as follows:

7 $$\begin{aligned} tfidf(t,d,D) = tf(t,d) \times idf(t,D) \end{aligned}$$

While TF-IDF can effectively identify terms that are important to a document within a corpus^30^, it lacks the ability to distinguish between categories. The objective of this method is to find terms that best differentiate one document from another, not terms that best differentiate one category from another. This limitation renders the high-dimensional features it generates unsuitable for direct use in the BRB models and, more critically, makes dimensionality reduction for classification purposes a challenging task. To overcome this limitation, this paper proposes the Discriminative TF-IDF method. This method embeds discriminative capability into the feature extraction process, ultimately generating low-dimensional and interpretable features that serve as input attributes for the BRB model, thereby perfectly aligning with the structural requirements of the BRB. The specific steps of the method are as follows:

Step 1: Data preprocessing. To eliminate noise and irrelevant information from the text data, the following procedures are applied to preprocess the raw text:

1. Removal of punctuation and digits.
2. Conversion of uppercase letters to lowercase.
3. Elimination of stop words.
4. Lemmatization.

Step 2: Initial vocabulary construction. First, a balanced spam set $D_{spam}$ and ham set $D_{ham}$ are extracted from the training dataset. Then, in contrast to the traditional TF-IDF method, an initial vocabulary *V* is constructed by applying TF-IDF vectorization to $D_{spam}$ only. Since spam often exhibits directed patterns, whereas ham is diverse and diffuse, learning features from the entire corpus would allow the feature space to be dominated by ham characteristics, thereby diluting the signals crucial for identifying spam. This spam-centric approach ensures that *V* captures terms that are characteristic of spam. *V* includes both unigrams and bigrams as features, since semantic intent in spam is often conveyed by specific word combinations. The IDF of each term in *V* is calculated based solely on $D_{spam}$, as defined below:

8 $$\begin{aligned} idf\left( t,D_{spam}\right) =log\frac{Z}{1+\left| \{d\in D_{spam}:t\in d\}\right| } \end{aligned}$$

Step 3: Calculation of average TF-IDF values of terms in ham and spam. For each $t \in V$, the TF-IDF value of *t* in *d* is defined as follows:

9 $$\begin{aligned} tfidf(t,d,D_{spam}) = tf(t,d) \times idf(t,D_{spam}) \end{aligned}$$

For each *t*, its average TF-IDF values across the two categories are defined as follows:

10 $$\begin{aligned} {avgti}_{spam}\left( t\right) =\frac{1}{Z_{spam}}\sum _{d\in D_{spam}}tfidf\left( t,d,D_{spam}\right) \end{aligned}$$

11 $$\begin{aligned} {avgti}_{ham}\left( t\right) =\frac{1}{Z_{ham}}\sum _{d\in D_{ham}}tfidf\left( t,d,D_{spam}\right) \end{aligned}$$

where $Z_{spam}$ and $Z_{ham}$ denote the total number of documents in $D_{spam}$ and $D_{ham}$.

Step 4: Calculation of spam tendency score. Based on the above steps, the spam tendency score $S\left( t\right)$ of *t* is defined as follows:

12 $$\begin{aligned} S\left( t\right) =\frac{{avgti}_{spam}\left( t\right) +\alpha }{{avgti}_{ham}\left( t\right) +\alpha } \end{aligned}$$

where $\alpha$ denotes a smoothing factor.

The smoothing factor $\alpha$ is introduced to prevent division by zero and to mitigate the influence of extreme score values. Considering the small-sample conditions of this study, $\alpha$ is initially set to 0.01 to maximally preserve the original data distribution patterns.

*S*(*t*) reflects the relative importance of *t* in spam compared to ham. $S(t)>1$ indicates that the term is more likely to appear in spam. $S(t)<1$ indicates that the term is more likely to appear in ham. $S(t)\approx 1$ indicates that the term is distributed similarly in both categories and has weak discriminative power.

Step 5: Ranking. All terms are ranked in descending order of *S*(*t*). Only terms satisfying $S(t)>1$ are retained, forming the final vocabulary $V^{*}$ along with their corresponding *S*(*t*).

Step 6: Calculation of input attribute values. Because the IDF of each term in *V* is calculated solely from $D_{spam}$, this design directly gives rise to two input attributes for the DTI-BRB model: the spam score (SS) and the spam keyword density (SKD). Using $V^{*}$, the SS and SKD of each document are computed as follows:

13 $$\begin{aligned} SS\left( d\right) =\sum _{t\in K_d} S\left( t\right) \end{aligned}$$

14 $$\begin{aligned} SKD\left( d\right) =\frac{\left| K_d\right| }{\left| V_d\right| } \end{aligned}$$

where $K_d$ denotes the set of terms from $V^*$ that occur in document *d*. $\left| K_d\right|$ denotes the number of terms in $K_d$. $\left| V_d\right|$ denotes the number of unique terms in *d*.

The calculated SS may fall outside the [0,1] range, while SKD lies within it. To ensure compatibility with the model’s inference process, both input attributes are normalized to the [0,1] range using min-max scaling.

Through the steps of the Discriminative TF-IDF method, SS and SKD are extracted to serve as the two input attributes of the DTI-BRB model. Although the selection of these input attributes is biased toward spam patterns, the BRB is capable of handling such uncertainty through its evidential reasoning mechanism. Furthermore, the subsequent optimization process fine-tunes the initial parameters, ensuring that this bias does not unnecessarily increase the misclassification of ham.

### The inference process of the DTI-BRB model

The inference process of the model follows the steps below:

Step 1: Calculate the matching degree between input attributes and reference values.

15 $$\begin{aligned} \alpha _i^k = {\left\{ \begin{array}{ll} \frac{x_i - A_i^l}{A_i^{l+1} - A_i^l}, & \text {if } A_i^k = A_i^l \\ \frac{A_i^{l+1} - x_i}{A_i^{l+1} - A_i^l}, & \text {if } A_i^k = A_i^{l+1} \\ 0, & \text {otherwise} \end{array}\right. } \end{aligned}$$

where $\alpha _i^k$ denotes the matching degree between the $i_{th}$ input attribute and its reference value in the $k_{th}$ rule. $x_i$ denotes the actual input value of the $i_{th}$ attribute. $A_i^k$ denotes the reference value of the $i_{th}$ attribute in the $k_{th}$ rule. $A_i^l$ and $A_i^{l+1}$ denote two adjacent reference values that satisfy $A_i^l\le x_i\le A_i^{l+1}$.

Step 2: Calculate rule activation weight.

16 $$\begin{aligned} \omega _k=\frac{\theta _k\prod _{i=1}^{M}\left( \alpha _i^k\right) ^{\delta _i}}{\sum _{l=1}^{K}\left[ \theta _l\prod _{i=1}^{M}\left( \alpha _i^l\right) ^{\delta _i}\right] } \end{aligned}$$

where $\omega _k$ denotes the activation weight of the $k_{th}$ rule. *M* denotes the total number of input attributes. *K* denotes the total number of rules.

Step 3: Rule fusion based on evidential reasoning. The fusion process begins by calculating the basic probability mass for each rule:

17 $$\begin{aligned} m_{n,l}=\omega _l\beta _{n,l} \end{aligned}$$

18 $$\begin{aligned} m_{D,l}=1-\omega _l\sum _{n=1}^{N}\beta _{n,l} \end{aligned}$$

where $m_{n,l}$ denotes the basic probability mass assigned to evaluation grade $D_n$. $m_{D,l}$ denotes the remaining unassigned probability mass in the $l_{th}$ rule. $\omega _l$ denotes the activation weight of the $l_{th}$ activated rule. $\beta _{n,l}$ denotes the belief degree of evaluation grade $D_n$ in the $l_{th}$ rule.

Subsequently, all rules are fused using the following formulas:

19 $$\begin{aligned} \beta _n = \frac{\mu \left[ \prod _{l=1}^{L} (m_{n,l} + m_{D,l}) - \prod _{l=1}^{L} m_{D,l}\right] }{1 - \mu \left[ \prod _{l=1}^{L} (1 - \omega _l)\right] }, \quad n = 1, 2, \ldots , N \end{aligned}$$

20 $$\begin{aligned} \mu =\left[ \sum _{n=1}^{N}\prod _{l=1}^{L}\left( m_{n,l}+m_{D,l}\right) -\left( N-1\right) \prod _{l=1}^{L}m_{D,l}\right] ^{-1} \end{aligned}$$

where $\beta _n$ denotes the combined belief degree of evaluation grade $D_n$ after fusion. *N* denotes the total number of evaluation grades. *L* denotes the total number of activated rules.

Step 4: Utility calculation.

21 $$\begin{aligned} u\left( S\left( x\right) \right) =\sum _{n=1}^{N}{u\left( D_n\right) \beta _n} \end{aligned}$$

where $u(D_n)$ denotes the utility value of evaluation grade $D_n$. *u*(*S*(*x*)) denotes the final output value of the model.

### The optimization process of the DTI-BRB model

The initial parameters of the DTI-BRB model are determined based on expert knowledge. However, due to the inherent ambiguity and limitations of prior knowledge, these parameters may lack sufficient accuracy. To obtain precise model parameters, this subsection introduces the P-CMA-ES algorithm for model optimization.

As an improved version of covariance matrix adaptation evolution strategy (CMA-ES), P-CMA-ES demonstrates significant advantages in handling constrained optimization problems^31^. Compared to standard CMA-ES, the primary strength of P-CMA-ES lies in its projection operation, which directly maps solutions to the feasible domain, thereby strictly satisfying equality constraints. This characteristic makes it particularly suitable for optimizing the parameters of the BRB. Furthermore, P-CMA-ES preserves all the advantages of CMA-ES, including derivative-free optimization, adaptive covariance matrix learning, and strong robustness. In the DTI-BRB model, the rule weights $\theta _k$, attribute weights $\delta _i$, and belief degrees $\beta _{n,k}$ require optimization. The optimization process is defined as follows:

22 $$\begin{aligned} \begin{aligned}&\min _{\Omega } \ MSE_{penalty}(\Omega ) = \frac{1}{N_t} \sum _{t=1}^{N_t} \left[ Penalty(y_t, \hat{y}_t) \cdot (\hat{y}_t - y_t)^2 \right] \\&\text {where } Penalty(y_t, \hat{y}_t) = {\left\{ \begin{array}{ll} 1.5, & \text {if } y_t = 1 \text { and } \hat{y}_t = 0 \\ 1.0, & \text {otherwise} \end{array}\right. } \\&\text {subject to: } 0 \le \theta _k \le 1,\quad 0 \le \delta _i \le 1,\quad 0 \le \beta _{n,k} \le 1, \\&\quad \sum _{n=1}^{2} \beta _{n,k} = 1,\quad \forall k = 1, \ldots , K;\ i = 1, \ldots , M;\ n = 1, 2 \end{aligned} \end{aligned}$$

where $\Omega$ denotes the set of parameters to be optimized. $N_t$ denotes the number of training samples. $y_t$ denotes the true label of the $t_{th}$ sample. $\widehat{y_t}$ denotes the model-predicted output of the $t_{th}$ sample.

**Fig. 2:**
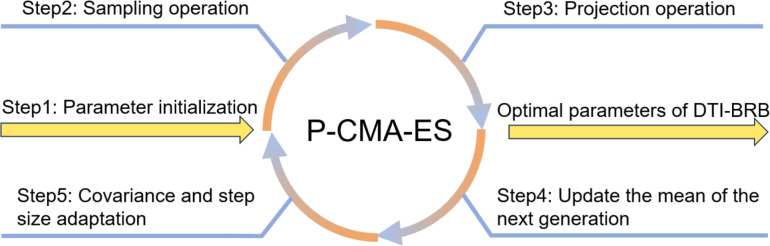
Optimization process.

The optimization process is illustrated in Fig. 2, with the steps as follows:

Step 1: Parameter initialization. Initialize the set of parameters to be optimized:

23 $$\begin{aligned} \begin{aligned}&\Omega ^{(0)} = \{\theta _1, \ldots , \theta _L, \beta _{1,1}, \beta _{2,1}, \ldots , \beta _{1,L}, \beta _{2,L}, \delta _1, \ldots , \delta _M\} \\&m^{(0)} = \Omega ^{(0)},\quad C^{(0)} = I,\quad \sigma ^{(0)}> 0,\quad p_c^{(0)} = 0,\quad p_\sigma ^{(0)} = 0 \end{aligned} \end{aligned}$$

where $\Omega ^{(0)}$ denotes the initial parameter vector. $m^{(0)}$ denotes the initial mean vector. $C^{(0)}$ denotes the initial covariance matrix. $\sigma ^{(0)}$ denotes the initial step size. $p_c^{(0)}$ and $p_\sigma ^{(0)}$ denote the initial evolution paths.

Step 2: Sampling operation. Generate a new population of candidate solutions based on the current distribution:

24 $$\begin{aligned} \Omega _c^{(g+1)} = m^{(g)} + \sigma ^{(g)} y_c,\quad y_c \sim N(0, C^{(g)}),\quad c = 1, \ldots , \lambda \end{aligned}$$

where *g* denotes the current generation. $\Omega _c^{\left( g+1\right) }$ denotes the $c_{th}$ candidate solution of the $\left( g+1\right) _{th}$ generation. $y_c$ denotes a random perturbation vector. $\lambda$ denotes the population size.

Step 3: Projection operation. Project each candidate solution onto the equality constraint surface to strictly satisfy all constraints. The projection is defined as follows:

25 $$\begin{aligned} \Omega _c^{(g+1)}(I) = \Omega _c^{(g+1)}(I) + \frac{1 - \sum _{j \in I} \Omega _c^{(g+1)}(j)}{|I|} \end{aligned}$$

where *I* denotes the set of variable indices involved in the same rule belief degree constraint. |*I*| denotes the number of variables in the equality constraint. $\Omega _c^{(g+1)}(I)$ denotes the components of candidate solution $\Omega _c^{(g+1)}$ corresponding to the index set *I*.

Step 4: Update the mean of the next generation. Evaluate the fitness of candidate solutions and update the mean of the search distribution:

26 $$\begin{aligned} m^{(g+1)} = \sum _{i=1}^{\mu } w_i \, \Omega _{i:\lambda }^{(g+1)} \end{aligned}$$

where $m^{(g+1)}$ denotes the updated mean vector of the distribution. $\mu$ denotes the number of parents. $w_i$ denotes the recombination weight. $\Omega _{i:\lambda }^{(g+1)}$ denotes the $i_{th}$ best solution in the $(g+1)_{th}$ generation based on fitness.

Step 5: Covariance and step size adaptation. Update the evolution paths and covariance matrix to adapt the search direction and step size:

27 $$\begin{aligned} C^{\left( g+1\right) }=\left( 1-c_1-c_\mu \right) C^{\left( g\right) }+c_1p_c^{\left( g+1\right) }\left( p_c^{\left( g+1\right) }\right) ^T+c_\mu \sum _{i=1}^{\mu }{w_iy_{i:\lambda }^{\left( g+1\right) }\left( y_{i:\lambda }^{\left( g+1\right) }\right) ^T} \end{aligned}$$

where $c_1$ and $c_\mu$ denote learning rates for the covariance matrix. $p_c^{(g+1)}$ denotes the updated evolution path. $y_{i:\lambda }^{(g+1)}$ denotes the normalized search step of the $i_{th}$ best solution.

Steps 2 to 5 are iteratively executed until the maximum number of evaluations is reached. The algorithm finally returns the optimized parameters $\Omega ^*$.

### Summary of the DTI-BRB modeling process

**Fig. 3:**
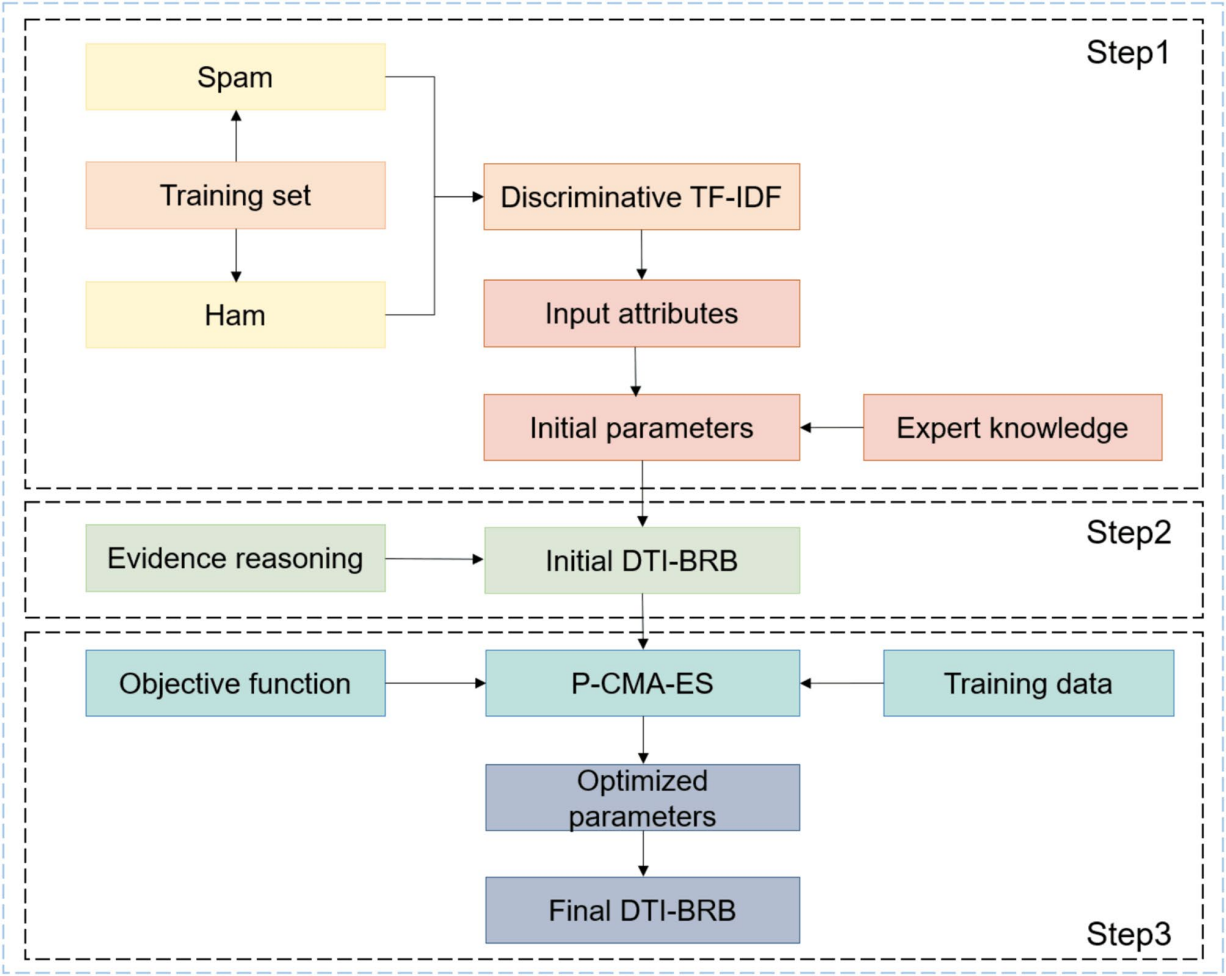
The modeling process of the DTI-BRB model.

Based on the preceding content, the modeling process of the DTI-BRB model is summarized in Fig. 3.

The steps are outlined as follows:

Step 1: Model parameter determination. Two input attributes are extracted via the Discriminative TF-IDF method. Based on this, the initial parameters of the DTI-BRB model are established by incorporating expert knowledge.

Step 2: Model construction. The inference process of the model adheres to the evidential reasoning mechanism described in the subsection "The inference process of the DTI-BRB model".

Step 3: Model optimization. Training data are fed into the model, and the mean squared error with a penalty term between the predicted output and the true labels is computed as the objective function. The P-CMA-ES optimization algorithm described in the subsection "The optimization process of the DTI-BRB model" is then employed to optimize the parameters of the DTI-BRB model.

## Case study

In this section, two case studies are conducted to validate the capability of the DTI-BRB model in dealing with scarce samples during the early stages of novel spam outbreaks. In the subsection "Validation of small-sample performance of the DTI-BRB model", the short message service (SMS) spam collection v.1 dataset is used to perform five-fold cross-validation experiments under different small-sample sizes. Additionally, an ablation experiment and comparative experiments are carried out to demonstrate the effectiveness of the model with limited data and to underscore the necessity of the Discriminative TF-IDF method. In the subsection "Validation of robustness and applicability of the DTI-BRB model", the fraud email dataset, a novel type of email data, is employed to verify the robustness and general applicability of the model.

### Validation of small-sample performance of the DTI-BRB model

#### SMS spam collection v.1 dataset

This study employs the SMS spam collection v.1 dataset, a labeled collection of text samples specifically compiled for mobile spam research (https://archive.ics.uci.edu/dataset/228/sms+spam+collection). This dataset contains 5,574 samples in total, comprising 4,827 ham and 747 spam. There are various types of spam, such as advertisements, phishing attempts, and prize-winning scams. First, data preprocessing was performed. Table 1 shows examples of the raw data, while Table 2 presents the corresponding preprocessed results.

**Table 1:** Dataset samples.

Label	Content
Ham	Even my brother is not like to speak with me. They treat me like aids patent.
Ham	I HAVE A DATE ON SUNDAY WITH WILL!!
Ham	Oh k...i’m watching here:)
Spam	Did you hear about the new ”Divorce Barbie”? It comes with all of Ken’s stuff!
Spam	88800 and 89034 are premium phone services call 08718711108

**Table 2:** Preprocessed samples.

Label	Content
Ham	Even brother like speak treat like aid patent
Ham	Date sunday
Ham	Oh watch
Spam	Hear new divorce barbie come ken stuff
Spam	Premium phone service call

For reliable evaluation and to avoid the impact of category imbalance on model performance evaluation, a balanced dataset was constructed. From each category of the original dataset, 100 samples were randomly selected for the experiment. Specifically, for each category, a randomization process was conducted separately using the random function in Excel to randomize the order of samples, after which the first 100 samples from the randomized list were taken. The relevant data are provided in Supplementary Table S1.

To comprehensively evaluate the predictive performance of the DTI-BRB model under small-sample conditions, four datasets with different sample sizes were constructed. The sample size *x* for each category was set to 20, 50, 80, and 100 respectively. To isolate the effect of sample size, a nested sampling approach was used. First, from the 100 samples in each category, the first 20, 50, 80, and all 100 samples were extracted as the experimental samples. This approach ensured that datasets with smaller *x* were strict subsets of those with larger *x*. When *x* increased from 20 to 50, changes in model performance could be attributed to the additional 30 samples. Finally, by merging the two categories of samples at the same *x* level, four datasets with total sample sizes of 40, 100, 160, and 200 were formed. This experiment aims to simulate typical scenarios ranging from extreme sample scarcity (*x*=20) to the upper limit of low-resource conditions (*x*=100), thereby enabling a detailed evaluation of the model’s detection performance across different small-sample sizes.

In this experiment, five-fold cross-validation was employed for each dataset to evaluate the stability of the DTI-BRB model. Each dataset was divided into five equal folds, with four of them used for training and the remaining one for testing. The experimental environment consisted of Windows 11 operating system and 12th Gen Intel(R) Core(TM) i9-12900H processor. Data preprocessing and feature extraction in the experiments were conducted in a Python environment, leveraging its powerful text-processing libraries for efficient feature computation. The resulting feature data were saved in Excel (.xlsx) format to serve as a standardized interface. Subsequently, as the code for the DTI-BRB model and the P-CMA-ES algorithm was developed in MATLAB, all models were trained and tested within the MATLAB environment to ensure environmental consistency.

#### Evaluation metrics

To evaluate the performance of the DTI-BRB spam detection model, four metrics were employed: accuracy, precision, recall, and F1-score. Since the focus was on spam detection, with ham as the background, spam was designated as the positive class and ham as the negative class. Accuracy measures the proportion of correctly classified instances among all samples. Precision represents the proportion of true positive instances among those predicted as positive. Recall indicates the proportion of actual positive instances that are successfully predicted as positive. F1-score is the harmonic mean of precision and recall. The formulas of accuracy, precision, recall, and F1-score are defined as follows:

28 $$\begin{aligned} accuracy=\frac{TP+TN}{TP+TN+FP+FN} \end{aligned}$$

29 $$\begin{aligned} precision=\frac{TP}{TP+FP} \end{aligned}$$

30 $$\begin{aligned} recall=\frac{TP}{TP+FN} \end{aligned}$$

31 $$\begin{aligned} F1-score=2\times \frac{precision\times recall}{precision+recall} \end{aligned}$$

where *TP* denotes true positives. *FP* denotes false positives. *TN* denotes true negatives. *FN* denotes false negatives.

#### Feature extraction

To transform textual data into numerical representations, the Discriminative TF-IDF method was applied to the raw sample data for feature extraction. The four small-sample datasets of different sizes were each divided into five folds. In five repeated experiments, four folds were used for training and one for testing. For each experiment, the following steps are performed:

First, the training set was separated into a spam set $D_{\textrm{spam}}$ and a ham set $D_{\textrm{ham}}$.

Second, a TF-IDF vectorizer was fitted on $D_{spam}$ to construct an initial vocabulary *V* and calculate the IDF value $idf(t,D_{spam})$ for each term *t*.

Then, as shown in equations (10) and (11), the average TF-IDF values of each *t* in *V* were computed. The spam tendency score *S*(*t*) for term *t* was calculated according to equation (12). After sorting the terms in descending order of *S*(*t*), only those with $S(t)>1$ were retained, forming the final vocabulary $V^*$ along with their corresponding *S*(*t*) values.

Finally, based on equations (13) and (14), SS and SKD were computed for each document.

**Fig. 4:**
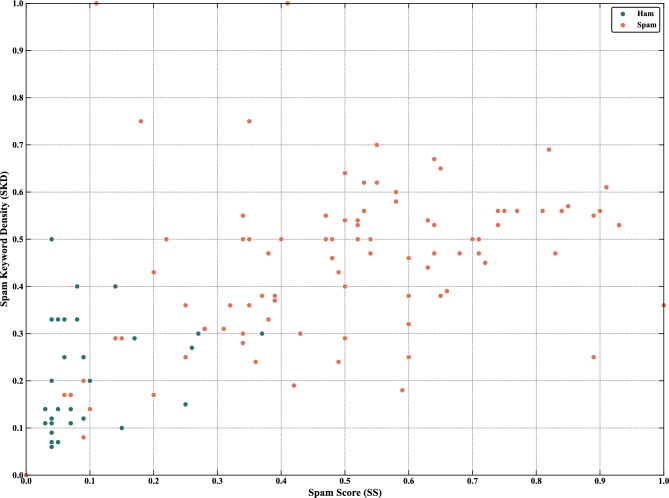
Distribution of 200 samples in the SS-SKD feature space (SMS spam collection v.1 dataset).

Figure 4 shows the distribution of 200 samples from the four small-sample datasets in the SS-SKD feature space. Ham samples are concentrated in the lower-left region, while most spam samples are distributed in the middle-upper region, indicating that the extracted features effectively separate ham from spam.

#### Construction of the DTI-BRB model

The DTI-BRB model takes SS and SKD as its two input attributes. As both attributes are considered equally important, their initial attribute weights are set to 0.5. In the BRB design, setting too few reference points for input attributes can lead to insufficient rule coverage, while too many may compromise inference efficiency. Therefore, both SS and SKD are discretized into four reference points: high (H), medium-high (MH), medium-low (ML), and low (L). The resulting 16 rules can adequately cover complex situations while ensuring good inference efficiency. The corresponding reference values for each point are determined based on expert knowledge, as summarized in Table 3.

The model output consists of two belief degrees: the confidence that a sample is ham (denoted as Y) and the confidence that it is spam (denoted as S). A sample is classified as spam if S exceeds Y. Initial rule weights and belief degrees are assigned according to expert knowledge, as detailed in Table 4. All initial rule weights are uniformly set to 1, indicating that no rule is considered more important than another. When both input attributes correspond to high reference points, the initial belief degree for spam is assigned a relatively high value. Conversely, when the input attributes correspond to conflicting reference points, the belief degrees for both ham and spam are initialized to approximately equal values. The subsequent P-CMA-ES optimization then fine-tunes these parameters. Table 5 shows the hyperparameters of the P-CMA-ES optimization algorithm implemented in this study.

**Table 3:** Reference points for SS and SKD.

Attributes	H	MH	ML	L
SS	0.51	0.28	0.05	0
SKD	0.56	0.39	0.08	0

**Table 4:** Initial rule weights and belief degrees.

Rule	Rule weight	SS	SKD	Belief degree
1	1	H	H	{0.05,0.95}
2	1	H	MH	{0.08,0.92}
3	1	H	ML	{0.15,0.85}
4	1	H	L	{0.25,0.75}
5	1	MH	H	{0.12,0.88}
6	1	MH	MH	{0.20,0.80}
7	1	MH	ML	{0.30,0.70}
8	1	MH	L	{0.45,0.55}
9	1	ML	H	{0.25,0.75}
10	1	ML	MH	{0.35,0.65}
11	1	ML	ML	{0.50,0.50}
12	1	ML	L	{0.65,0.35}
13	1	L	H	{0.50,0.50}
14	1	L	MH	{0.65,0.35}
15	1	L	ML	{0.85,0.15}
16	1	L	L	{0.95,0.05}

**Table 5:** Hyperparameters of the P-CMA-ES optimization algorithm.

Hyperparameter	Value
Initial step size	0.3
Population size lower bound	20
Population size scaling factor	4
Parent population ratio	0.5
Rank-one update learning rate coefficient	2.5
Step size damping base value	0.8
Mirror point perturbation coefficient	0.1
Maximum number of evaluations	200

#### Results analysis

**Fig. 5:**
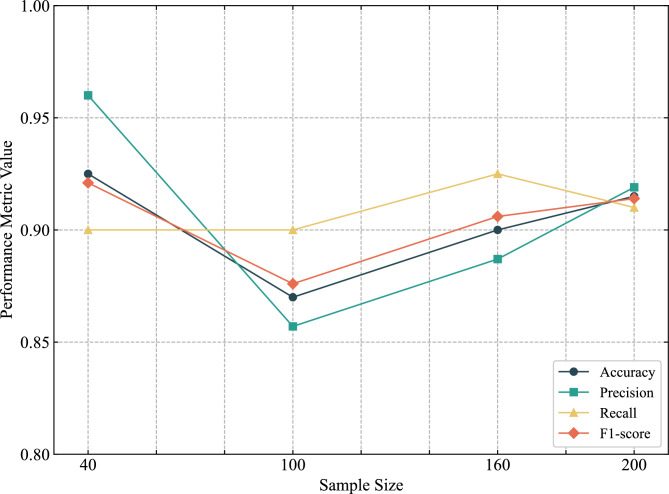
Performance of the DTI-BRB model on different sample sizes.

Figure 5 shows the average evaluation metrics of the DTI-BRB model across different sample sizes, derived from five-fold cross-validation. When the sample size increased from 40 to 100, accuracy, precision, and F1-score all exhibited a certain degree of decline. One possible explanation is that with only 8 samples in the test set at the sample size of 40, the sample characteristics might have been simplistic. As the sample size increased to 100, the growing complexity of data distribution likely contributed to performance fluctuations.

As the sample size increased further to 160 and 200, all metrics recovered and stabilized. This indicates that the performance of the DTI-BRB model improved and converged to a stable level with more training data. At 200 samples, all metrics reached or exceeded 91%. These results demonstrate that in small-sample conditions, the DTI-BRB model exhibits excellent data utilization efficiency. With only 200 samples, the model can learn stable and effective features.

**Fig. 6:**
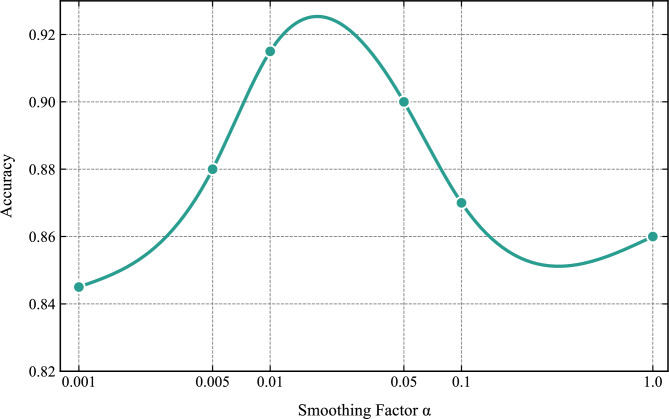
The trend of accuracy with respect to the smoothing factor $\alpha$.

In this experiment, $\alpha$ in equation (12) was set to 0.01. To verify the rationality of this initial value and evaluate the impact of $\alpha$ on model performance, the classification performance was further compared for the $\alpha$ values of {0.001, 0.005, 0.05, 0.1, 1.0}. Based on 200 samples, Fig. 6 shows the trend of accuracy with respect to $\alpha$. Within the range of $\alpha$ = 0.005 to 0.05, the classification performance remained high and stable, indicating that the model performance is insensitive to minor adjustments of $\alpha$ in this interval. Specifically, the model yielded superior performance when $\alpha$ was set to 0.01, which validates the rationality of the initial value selection. For the sake of experimental consistency, $\alpha$ was fixed at 0.01 in all subsequent experiments.

#### Ablation experiment

To validate the effectiveness of discriminative features for dimensionality reduction, an ablation experiment was designed. Using the DTI-BRB model as the baseline, the discriminative capability was removed from the feature extraction process, resulting in a new model referred to as TI-BRB. The feature extraction steps for the TI-BRB model are as follows:

First, all the document collection *D* was used to fit a TF-IDF vectorizer, constructing *V* that included both unigrams and bigrams, and calculating the IDF values $\textrm{idf}(t,D)$. Then, from *V*, terms that appear in $D_{\textrm{spam}}$ were filtered to form $V^*$. Finally, for each document *d*, SS was computed as $\sum _{t \in K_d} \textrm{tfidf}(t, d, D)$, and SKD was computed according to equation (14).

**Fig. 7:**
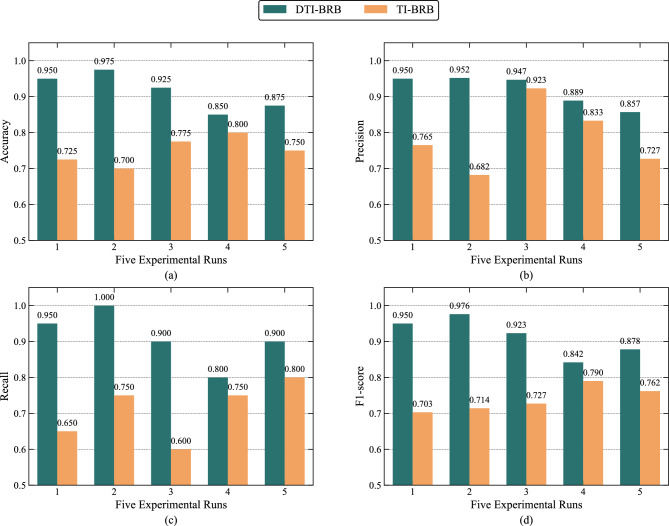
Performance comparison between DTI-BRB and TI-BRB (sample size = 200). (a) Accuracy. (b) Precision. (c) Recall. (d) F1-score.

Five-fold cross-validation is conducted using the same training and test sets as those employed in the DTI-BRB model. Figure 7 shows the results of all five experimental runs for both DTI-BRB and TI-BRB at a sample size of 200. The results indicate that DTI-BRB significantly outperforms TI-BRB across all metrics, demonstrating that the discriminative capability is a key factor for performance improvement. As this capability constitutes the core of the Discriminative TF-IDF method, this experiment validates the method’s effectiveness.

#### Comparative study

To comprehensively demonstrate the superiority of the DTI-BRB spam detection model, we selected a range of representative models for comparison: traditional machine learning models, including k-nearest neighbor (KNN)^32^, backpropagation neural network (BPNN)^33^, radial basis function (RBF)^34^, and ELM^35^; deep learning models, including CNN^36^ and MLP^37^; and fuzzy system-based methods, including FSVM^38^ and fuzzy neural network (FNN)^39^. All models were evaluated using five-fold cross-validation to ensure a comprehensive assessment. The hyperparameter settings for all comparison models are presented in Table 6. Table 7 presents the average accuracy, precision, recall, and F1-score achieved by each model on datasets of different sample sizes.

**Table 6:** Hyperparameter settings of comparison models.

Model	Hyperparameter settings
KNN	k = 3, euclidean distance, equal weights
BPNN	hidden neurons: 10, epochs: 200, learning rate: 0.05, target error: 0.0001
RBF	spread constant: 0.5, max neurons: 50, target error: 0.01
ELM	hidden neurons: 50, activation: sigmoid, output: softmax
CNN	convolutional kernels: 8, FC neurons: 16, dropout: 0.5, max epochs: 50, batch size: 16, learning rate: 0.001, L2: 0.01
FSVM	c: 1.0, RBF kernel $\alpha$: 0.707, membership scale: 1.0, max repetitions: 5
FNN	fuzzy sets: 3, hidden neurons: 6, learning rate: 0.01, max epochs: 100
MLP	hidden layers: [16, 8, 4], dropout: [0.4, 0.3, 0.2], max epochs: 80, batch size: 8, learning rate: 0.001

**Table 7:** Performance comparison of different models across four sample sizes.

Model	Sample size	Accuracy	Precision	Recall	F1-score
DTI-BRB	40	0.925 (± 0.061)	0.960 (± 0.089)	0.900 (± 0.129)	0.921 (± 0.066)
	100	0.870 (± 0.062)	0.857 (± 0.092)	0.900 (± 0.062)	0.876 (± 0.049)
	160	0.900 (± 0.056)	0.887 (± 0.077)	0.925 (± 0.037)	0.906 (± 0.046)
	200	0.915 (± 0.049)	0.919 (± 0.040)	0.910 (± 0.068)	0.914 (± 0.049)
KNN	40	0.925 (± 0.061)	0.960 (± 0.089)	0.900 (± 0.129)	0.921 (± 0.066)
	100	0.860 (± 0.076)	0.859 (± 0.104)	0.880 (± 0.067)	0.869 (± 0.062)
	160	0.844 (± 0.077)	0.827 (± 0.082)	0.875 (± 0.062)	0.850 (± 0.064)
	200	0.890 (± 0.064)	0.893 (± 0.064)	0.890 (± 0.064)	0.891 (± 0.064)
BPNN	40	0.900 (± 0.112)	0.933 (± 0.149)	0.900 (± 0.129)	0.874 (± 0.089)
	100	0.790 (± 0.050)	0.817 (± 0.114)	0.780 (± 0.084)	0.789 (± 0.039)
	160	0.825 (± 0.078)	0.844 (± 0.086)	0.819 (± 0.114)	0.821 (± 0.075)
	200	0.875 (± 0.045)	0.866 (± 0.052)	0.890 (± 0.045)	0.877 (± 0.033)
RBF	40	0.925 (± 0.061)	0.960 (± 0.089)	0.900 (± 0.129)	0.921 (± 0.066)
	100	0.740 (± 0.093)	0.787 (± 0.130)	0.780 (± 0.164)	0.741 (± 0.089)
	160	0.794 (± 0.128)	0.845 (± 0.173)	0.788 (± 0.125)	0.798 (± 0.133)
	200	0.620 (± 0.173)	0.619 (± 0.150)	0.840 (± 0.031)	0.703 (± 0.125)
ELM	40	0.850 (± 0.112)	0.933 (± 0.149)	0.850 (± 0.212)	0.804 (± 0.112)
	100	0.680 (± 0.062)	0.758 (± 0.142)	0.620 (± 0.187)	0.630 (± 0.142)
	160	0.825 (± 0.056)	0.839 (± 0.077)	0.813 (± 0.084)	0.822 (± 0.051)
	200	0.785 (± 0.092)	0.810 (± 0.088)	0.760 (± 0.118)	0.773 (± 0.098)
CNN	40	0.900 (± 0.112)	1.000 (± 0.000)	0.800 (± 0.204)	0.876 (± 0.131)
	100	0.820 (± 0.028)	0.900 (± 0.103)	0.740 (± 0.089)	0.804 (± 0.025)
	160	0.844 (± 0.092)	0.865 (± 0.072)	0.813 (± 0.136)	0.834 (± 0.103)
	200	0.860 (± 0.046)	0.913 (± 0.061)	0.800 (± 0.089)	0.849 (± 0.053)
FSVM	40	0.925 (± 0.070)	1.000 (± 0.000)	0.850 (± 0.141)	0.914 (± 0.064)
	100	0.800 (± 0.050)	0.885 (± 0.125)	0.720 (± 0.111)	0.783 (± 0.051)
	160	0.850 (± 0.090)	0.858 (± 0.078)	0.838 (± 0.127)	0.845 (± 0.097)
	200	0.864 (± 0.039)	0.902 (± 0.045)	0.820 (± 0.075)	0.857 (± 0.046)
FNN	40	0.900 (± 0.056)	0.960 (± 0.089)	0.850 (± 0.125)	0.892 (± 0.049)
	100	0.810 (± 0.075)	0.822 (± 0.105)	0.820 (± 0.100)	0.812 (± 0.062)
	160	0.869 (± 0.062)	0.859 (± 0.069)	0.887 (± 0.050)	0.872 (± 0.048)
	200	0.880 (± 0.077)	0.895 (± 0.067)	0.870 (± 0.083)	0.876 (± 0.076)
MLP	40	0.950 (± 0.069)	1.000 (± 0.000)	0.900 (± 0.125)	0.943 (± 0.064)
	100	0.810 (± 0.040)	0.881 (± 0.119)	0.760 (± 0.089)	0.796 (± 0.041)
	160	0.888 (± 0.061)	0.899 (± 0.072)	0.875 (± 0.059)	0.886 (± 0.054)
	200	0.885 (± 0.054)	0.905 (± 0.047)	0.860 (± 0.060)	0.882 (± 0.053)

**Fig. 8:**
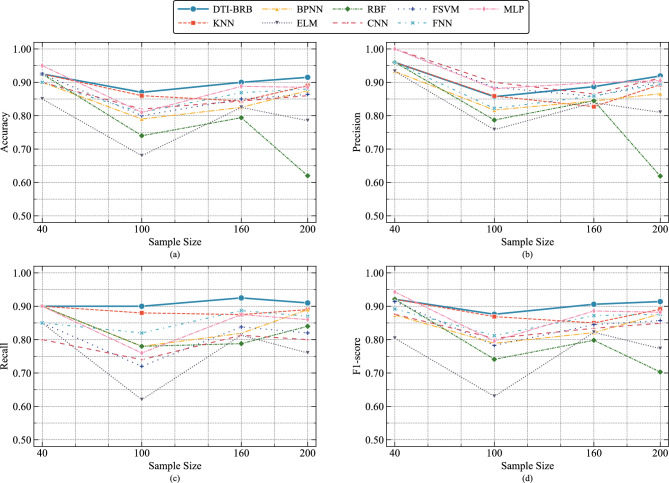
Performance trends of different models across four sample sizes. (a) Accuracy. (b) Precision. (c) Recall. (d) F1-score.

To present the tabular data clearly, Fig. 8 shows the performance trends of each model as the sample size increases. The performance of all models declined as the sample size expanded from 40 to 100, with the MLP, FSVM, and BPNN exhibiting the most significant drops. Across the increase in sample size from 100 to 200, the DTI-BRB model consistently achieved the best and most stable performance. The KNN, FNN, and CNN demonstrated relatively good stability, though their overall performance was slightly lower than that of the DTI-BRB model. In contrast, the RBF and ELM were the most sensitive to changes in sample size, particularly the RBF model, which showed severe performance degradation with 200 samples, indicating poor stability.

### Validation of robustness and applicability of the DTI-BRB model

#### Fraud email dataset

To validate the robustness and applicability of the DTI-BRB model, the fraud email dataset^40^ was selected for further investigation. This dataset is publicly accessible via https://www.kaggle.com/datasets/llabhishekll/fraud-email-dataset. It contains 11,948 samples in total, comprising 6,744 ham and 5,204 spam. The spam in this dataset are predominantly focused on financial or commercial fraud, which is a specific type not covered by the SMS spam collection v.1 dataset, and tend to be longer than the ham.

Since experiments on the SMS spam collection v.1 dataset under different small-sample sizes had shown that the performance of the DTI-BRB model improved and stabilized as the sample size increased, a representative size of 200 samples was adopted for this experiment. This choice aimed to verify whether the model remains effective when encountering a new type of spam while still under small-sample conditions. First, data preprocessing was performed. Then, 100 samples were randomly selected from each category of the original dataset for the experiment. Specifically, for each category, a randomization process was conducted separately using the random function in Excel to randomize the order of samples, after which the first 100 samples from the randomized list were taken. The relevant data are provided in Supplementary Table S2.

The data preprocessing, the feature extraction, the evaluation metrics, and the evaluation method (5-fold cross-validation) remained consistent with the previous experiments. To further assess the robustness of the model, the initial parameter settings of the DTI-BRB model and comparison models were kept identical to those used earlier.

**Fig. 9:**
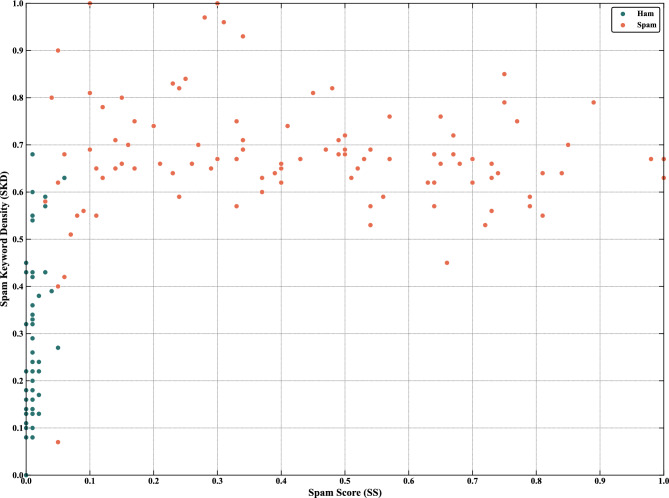
Distribution of 200 samples in the SS-SKD feature space (fraud email dataset).

Figure 9 shows the distribution of 200 samples in the SS-SKD feature space. It can be observed that the Discriminative TF-IDF method effectively separates ham and spam. However, the distribution differs substantially from that of the SMS spam collection v.1 dataset, with the SKD of the ham generally exhibiting medium-high or high.

#### Results analysis

Through five-fold cross-validation, the DTI-BRB model achieved an average accuracy of 95.5%, an average precision of 94.5%, an average recall of 97.0%, and an average F1-score of 95.6%. This robust performance can be attributed to the rule-based architecture of the model. Although some ham contain a relatively high proportion of spam keywords, two rules within the 16-rule system of the DTI-BRB model provide clear initial belief degree assignments to handle this scenario, intuitively demonstrating the interpretable decision logic of the model:

Rule 13: if the reference point of SS is L and the reference point of SKD is H, then the initial belief degree for classifying the sample as ham is 0.50 and as spam is 0.50.

Rule 14: if the reference point of SS is L and the reference point of SKD is MH, then the initial belief degree for classifying the sample as ham is 0.65 and as spam is 0.35.

Though feature distributions vary considerably, they remain within the coverage scope of the DTI-BRB rule system, enabling the model to maintain good performance without altering its initial parameters. Building on this, the P-CMA-ES optimization algorithm further enhances performance by fine-tuning rule weights and belief degrees while preserving the core rule structure. This allows the model to quickly adapt to new email types without structural modifications. Consequently, by leveraging its belief rule-based architecture, the DTI-BRB model demonstrates an ability to learn efficiently from limited data and generalize well to novel data types, which are precisely the strengths needed to address the scarcity of samples during the early stages of emerging spam outbreaks.

#### Comparative study

Table 8 shows the performance of all models. In this dataset, the initial parameters of all models remain consistent with those in the previous experiments. Under this condition, only the DTI-BRB model maintains stable performance, indicating that the other models are unable to adapt to the new type of spam without adjusting their initial parameters. Most of these comparison models exhibit high recall but low precision, indicating that while they can effectively capture and identify spam, they simultaneously misclassify a substantial number of ham as spam. This phenomenon arises because spam features are relatively homogeneous and thus easier for models to learn, enabling high recall. However, these models lack the discriminative capacity to handle the greater variability within ham. Crucially, they fail to incorporate expert knowledge, such as the insight that a combination of low SS and high SKD may correspond to a ham, leading to low precision. Although FSVM, FNN, and DTI-BRB are all interpretable models, the interpretability of FSVM and FNN is largely confined to aspects like input fuzzification or feature importance, and their performance in this task remains suboptimal.

**Table 8:** Performance comparison of different models with 200 samples.

Model	Accuracy	Precision	Recall	F1-score
DTI-BRB	0.955 (± 0.029)	0.945 (± 0.049)	0.970 (± 0.042)	0.956 (± 0.027)
KNN	0.830 (± 0.092)	0.783 (± 0.131)	0.950 (± 0.039)	0.853 (± 0.066)
BPNN	0.860 (± 0.046)	0.801 (± 0.070)	0.970 (± 0.027)	0.875 (± 0.031)
RBF	0.870 (± 0.072)	0.904 (± 0.097)	0.840 (± 0.122)	0.864 (± 0.084)
ELM	0.855 (± 0.022)	0.851 (± 0.081)	0.880 (± 0.097)	0.858 (± 0.020)
CNN	0.820 (± 0.070)	0.764 (± 0.116)	0.960 (± 0.045)	0.845 (± 0.048)
FSVM	0.825 (± 0.064)	0.769 (± 0.113)	0.960 (± 0.045)	0.849 (± 0.048)
FNN	0.855 (± 0.093)	0.823 (± 0.143)	0.950 (± 0.042)	0.874 (± 0.073)
MLP	0.825 (± 0.064)	0.769 (± 0.113)	0.960 (± 0.045)	0.849 (± 0.048)

Among the four evaluation metrics, the F1-score is selected as the core indicator for assessing the overall performance of spam detection models. To evaluate the statistical significance of the performance advantage of the DTI-BRB model with 200 samples, we conducted hypothesis testing based on the five F1-scores obtained from 5-fold cross-validation.

Given the limited number of observations (n=5), which makes it difficult to satisfy the normality assumptions required by parametric tests, we employed the non-parametric paired Wilcoxon signed-rank test (two-tailed, alpha level=0.05). Since the DTI-BRB model was compared against eight comparison models, the Benjamini-Hochberg (BH) correction was applied to the obtained p-values to control the false discovery rate from multiple comparisons.

As shown in Table 9, the DTI-BRB model consistently demonstrates positive performance differences against all comparison models ($\Delta$F1 ranging from +0.0805 to +0.1104). The exceptionally large effect sizes (r > 0.78, with most exceeding 0.90) far surpass the conventional threshold for large effects (r $\ge$ 0.5). Furthermore, all 95% confidence intervals exclude zero, indicating that the observed advantages are unlikely to be zero. While the BH-corrected p-values (0.0714–0.1250.0714.1250) slightly exceed the conventional 0.05 threshold, this reflects the limited statistical power with only five observations. Nevertheless, the combination of large effect sizes, consistent directionality, and confidence intervals excluding zero provides compelling substantive evidence for the performance advantage of the DTI-BRB model. Future work will extend the DTI-BRB model to more diverse spam datasets with larger-scale experiments to further validate these findings.

**Table 9:** Statistical comparison between the DTI-BRB model and comparison models.

Comparison model	Mean difference ($\Delta$F1)	Effect size (r)	95% CI	BH-corrected p-value
KNN	+0.1028	0.9045	[0.0438, 0.1895]	0.0714
BPNN	+0.0805	0.9045	[0.0473, 0.1222]	0.0714
RBF	+0.0916	0.9045	[0.0300, 0.1692]	0.0714
ELM	+0.0978	0.9045	[0.0737, 0.1215]	0.0714
CNN	+0.1104	0.9045	[0.0629, 0.1653]	0.0714
FSVM	+0.1069	0.9045	[0.0629, 0.1617]	0.0714
FNN	+0.0817	0.7839	[0.0174, 0.1624]	0.1250
MLP	+0.1069	0.9045	[0.0629, 0.1617]	0.0714

## Conclusion

To address the dual challenges of sample scarcity and opaque decision-making in the early stages of novel text-based spam outbreaks, the DTI-BRB spam detection model is proposed in this paper. To accommodate the high-dimensional nature of textual data, the Discriminative TF-IDF feature extraction method is designed, effectively ensuring the feasibility of the DTI-BRB model. Comparative experiments with traditional machine learning models, deep learning models, and fuzzy system-based methods at various small-sample scales further validate the superior robustness and generalization capability of the proposed DTI-BRB model under limited data conditions. The intrinsic interpretability of the DTI-BRB model not only enhances the credibility of the results but also provides experts with the means to understand emerging spam features, verify model logic, and implement rapid interventions. The DTI-BRB model primarily focuses on spam detection at the textual content level. Future research will explore the integration of the DTI-BRB model with methods for handling non-textual features, such as images and URLs, and apply it to larger-scale experiments.

## Supplementary Information

Supplementary Information.

## Data Availability

The public datasets used in this research can be found at the following links: https://archive.ics.uci.edu/dataset/228/sms+spam+collection and https://www.kaggle.com/datasets/llabhishekll/fraud-email-dataset.
