# Supplementary material for: A spam detection model based on the discriminative TF-IDF belief rule base
Source: Sci Rep. 2026 Mar 4;16:11962. doi: 10.1038/s41598-026-42223-6 (PMC13069011; doi:10.1038/s41598-026-42223-6)
Supplement: Supplementary file 1 — Supplementary Information. [file 41598_2026_42223_MOESM1_ESM.pdf]

Supplementary Table S1

0	call later network urgnt sm
0	nope wait sch daddy
0	well gon na finish bath good fine night
0	bring home wendy
0	sir wait mail
0	come mu sort narcotic situation
0	stuff sell tell
0	aight hit get cash
0	message text miss sender name miss number miss send date miss miss lot thats everything miss send via fullonsms com
0	well imma definitely need restock thanksgiv let know
0	take exam march
0	hi frnd good way avoid missunderstd wit belov one
0	necessarily expect do get back though headin
0	log what sdryb
0	long since scream princess
0	jos ask wana meet
0	ya srsly good yi tho
0	lol always convince
0	perform
0	haha mayb rite know well da feel like someone gd lor faster go find one gal group attach liao
0	pete please ring meive hardly gotany credit
0	mum repent
0	do hand celebration full swing yet
0	operate lt gt
0	depends quality want type sent boye fade glory want ralphs maybe
0	come pick come immediately aft lesson
0	thanks pick trash
0	chance might evaporate soon violated privacy steal phone number employer paperwork cool please contact report supervisor
0	get lt gt way could pick
0	interested like
0	sir need axis bank account bank address
0	thats bite weird even suppose happen good idea sure pub
0	meet call later
0	hi baby im cruisin girl friend give call hour home thats alright fone fone love jenny xxx
0	okies go yan jiu skip ard oso go cine den go mrt one blah blah blah
0	sorry roommate take forever ok come
0	bruv hope great break rewarding semester
0	ok anyway need change say
0	hi da today class
0	pick another th do
0	call message miss call

0	please give connection today lt decimal gt refund bill
0	whatsup dont want sleep
0	respond
0	hi hope get txt journey hasnt gd min late think
0	probably gon na see later tonight lt
0	go jurong point crazy available bugis great world la buffet cine get amore what
0	place man
0	hi wk ok hols yes bite run forget hairdresser appointment four need get home shower beforehand cause prob
0	see letter car
0	thank
0	go thru different feel wavering decision cop individual time heal everything believe
0	lover need
0	cool text ready
0	ok tell stay yeah tough optimistic thing improve month
0	sorry call later ok bye
0	predict what time finish buying
0	sure make sure know smokin yet
0	oh send address
0	headin towards busetop
0	nah think go usf life around though
0	usf guess might well take car
0	aathi dear
0	co want thing
0	per request melle melle oru minnaminunginte nurungu vettam set callertune caller press copy friend callertune
0	hop enjoy game yesterday sorry touch pls know fondly bein thot great week abiola
0	aiyo always ex one dunno abt mei reply first time reply fast lucky workin huh get bao sugardad ah gee
0	geeeee love much barely stand
0	fine imma get drink somethin want come find
0	call meet
0	yeah hopefully tyler could maybe ask around bit
0	macha dont feel upset assume mindset believe one even wonderful plan let life begin call anytime
0	print oh lt gt come upstairs
0	also dont msg reply msg
0	still look car buy go driving test yet
0	yes think office lap room think thats last day didnt shut
0	gon na pick burger way home even move pain killing
0	cant think anyone spare room top head
0	hello good week fancy drink something later
0	idc get weaseling way shit twice row
0	delete contact
0	ok may free gym
0	good afternoon sunshine dawn day refresh happy alive breathe air smile think love always
0	tire argue week week want

0	sea lay rock rock envelope envelope paper paper word
0	stop however suggest stay someone able give or every stool
0	happy new year dear brother really miss get number decide send text wish happiness abiola
0	jay say double faggot
0	come room point iron plan weekend
0	dunno jus say go lido time
0	hurt tease make cry end life die plz keep one rise grave say stupid miss nice day bslvyl
0	tell accenture confirm true
0	great
0	aight yo dat straight dogg
0	go try month ha ha joke
0	special
0	luton ring around
0	say good sign well know track record reading woman
0	famous quote develop ability listen anything unconditionally without lose temper self confidence mean married
0	yo guy ever figure much need alcohol jay try figure much safely spend weed
1	ou guarantee late nokia phone gb ipod mp player prize txt word collect ibhltd ldnw mtmsgrcvd
1	claire havin borin time alone wan na cum nite chat hope luv claire xx call minmoremobsemspobox po wa
1	today voda number end select receive award hava match please call quote claim code standard rate app
1	dear subscriber draw gift voucher enter receipt correct ans elvis presley birthday txt answer
1	important customer service announcement premier call freephone
1	welcome uk mobile date msg free give free call future mg bill daily cancel send go stop
1	hi ibh customer loyalty offer new nokia mobile txtauction txt word start get
1	value customer pleased advise follow recent review mob award bonus prize call
1	receive mobile content enjoy
1	win new harry potter order phoenix book reply harry answer question chance first among reader
1	free tone hope enjoy new content text stop unsubscribe help provide tone co uk
1	select stay top british hotel nothing holiday value dial claim national rate call bx sw
1	twinks bear scallies skin jock call miss weekend fun call min stop text call nat rate
1	urgent try contact today draw show prize guarantee call land line claim valid hr
1	text banneduk see cost textoperator ga xxx
1	private account statement show un redeem point call identifier code xx expire
1	urgent nd attempt contact prize yesterday still await collection claim call acl pm
1	dear voucher holder claim st class airport lounge pass use holiday voucher call booking quote st class
1	cash balance currently pound maximize cash send go msg cc po box tcr
1	send logo lover name join heart txt love name name mobno eg love adam eve yahoo pobox wq txtno ad
1	nokia tone mob every week txt nok st tone free get txtin tell friends tone reply hl info
1	thanks ringtone order reference charge gbp per week unsubscribe anytime call customer service
1	congrats year special cinema pas call suprman matrix starwars etc free bx ip pm dont miss
1	today offer claim worth discount voucher text yes savamob member offer mobile sub unsub reply
1	hi lookin saucy daytime fun wiv busty marry woman free next week chat sort time janinexx call minmobsmorelkpobox hp fl
1	free message activate free text message reply message word free term condition visit
1	want cock hubby away need real man satisfy txt wife string action txt stop end txt rec ea otbox la

1	fantasy football back tv go sky gamestar sky active play dream team score start saturday register sky opt
1	loan purpose homeowner tenant welcome previously refuse still help call free text back help
1	call freephone
1	sm service inclusive text credit pls goto login unsubscribe stop extra charge help po box ip
1	day euro kickoff keep informed late news result daily unsubscribe send get euro stop
1	private account statement xxxxxx show un redeem point call identifier code expires
1	urgent mobile bonus caller prize nd attempt reach call asap box qp ppm
1	urgent try contact today draw show prize guarantee call land line claim valid hr
1	voucher marsms log onto discount credit opt reply stop customer care call
1	thanks ringtone order ref number mobile charge tone arrive please call customer service
1	hi night lucky night uve invite xchat uks wild chat txt chat msgrcvdhg suite land row hl ldn yr
1	stop club tone reply stop mix see tone com enjoy html term club tone cost gbp week mfl po box mk wt
1	reminder download content already pay goto mymoby tv collect content
1	account xxxxxxxxx show unredeem point claim call identifier code xxxxx expires
1	hi lucy hubby meetins day fri alone hotel fancy cumin pls leave msg day lucy call minmobsmorelkpobox hp fl
1	sms auction nokia get win free auction take part send nokia hg suite land row jhl
1	one register subscriber enter draw gift voucher reply enter unsubscribe text stop
1	free entry weekly comp chance win ipod txt pod get entry std txt rate apply detail
1	registered subscriber yr draw gift voucher enter receipt correct ans next olympics txt an
1	urgent mobile award bonus caller prize nd attempt contact call box qu bt national rate
1	auction round high bid next maximum bid bid send bid bid good luck
1	moby pub quiz win high street prize know new duchess cornwall txt first name unsub stop sp
1	ever notice drive anyone go slow idiot everyone drive faster maniac
1	loan purpose homeowner tenant welcome previously refuse still help call free text back help
1	award city break could win summer shopping spree every wk txt store skilgme tscs winawk age perwksub
1	romcap1 everyone around respond well presence since warm outgo bring real breath sunshine
1	eerie nokia tone rply tone title eg tone dracula title ghost addamsfa munsters exorcist twilight pobox wq
1	thanks ringtone order reference number mobile charge tone arrive please call customer service
1	thanks ringtone order reference number mobile charge tone arrive please call customer service colour red text colour txtstar
1	network operator service free visit biz
1	come take little time child afraid dark become teenager want stay night
1	polyphonic tone mob every week txt pt st tone free get txtin tell friends tone reply hl info
1	rct thnq adrian text rgds vatian
1	get st ringtone free reply msg tone gr top tone phone every week per wk opt send stop
1	congratulation award either yrs supply cd virgin record mystery gift guarantee call pm approx min
1	today vodafone number end last four digit select receive award number match please call claim award
1	guarantee award even cashto claim award call free stop getstop php
1	mobile month entitle update late colour mobile camera free call mobile update co free
1	final chance claim worth discount voucher today text yes savamob member offer mobile savamob pobox uz sub
1	important customer service announcement premier
1	hot live fantasy call per min ntt ltd po box croydon cr wb
1	hi customer loyalty offer new nokia mobile txtauction txt word start get ctxt tc mtmsg
1	urgent mobile award bonus caller prize nd attempt contact call box qu

1	great new offer double min double txt good orange tariff get late camera phone free call mobileupd free stoptxt
1	customer loyalty offer new nokia mobile txtauction txt word start get ctxt tc mtmsg
1	network allow company bill sms responsible supplier shop give guarantee sell
1	free day sexy st george day pic jordan txt pic dont miss every wk saucy celeb pic pocketbabe co uk wk
1	reply name address receive post week completely free accommodation various global location ph
1	december mobile mths entitle update late colour camera mobile free call mobile update vco free
1	go ba0as callfreefone speak live operator claim either ba0as cruise cash opt txt
1	sms auction brand new nokia auction today auction free join take part txt nokia hg suite land row hl
1	forwarded please call immediately urgent message wait
1	free entry wkly comp win fa cup final tkts st may text fa receive entry question std txt rate apply
1	congratulation week competition draw prize claim call stop sm ppm
1	lookatme thanks purchase video clip lookatme charge think good send video mmsto
1	forward free entry weekly comp send word enter
1	take part mobile survey yesterday text use however wish get txts send txt
1	free top ringtone sub weekly ringtone get st week free send subpoly per week stop sm
1	contract mobile mnths late motorola nokia etc free double min text orange tariff text yes callback remove record
1	msg mobile content order resent previous attempt fail due network error query
1	hear new divorce barbie come ken stuff
1	want new video handset time network min unlimited text camcorder reply call del sit
1	please call customer service representative freephone pm guarantee cash prize
1	try contact reply offer video phone anytime network mins half price line rental camcorder reply call
1	new mobile must go txt nokia collect today optout gbp mtmsg txtauction
1	six chance win cash pound txt csh send cost day day tsandcs apply reply hl info
1	congrats mobile videophones call videochat wid mate play java game dload polyph music noline rentl bx ip
1	congratulation thanks good friend xmas prize claim easy call per minute bt national rate
1	know know send chat let find msg rcvd hg suite land row hl ldn year
1	text pas collect polyphonic ringtones normal gprs charge apply enjoy tone
1	cash prize claim call
1	private account statement show un redeem point call identifier code expires
1	final chance claim worth discount voucher today text yes savamob member offer mobile savamob pobox uz sub

Supplementary Table S2

0	agree also folk need hosp care period several thing like consider sentvia blackberry
0	draft
0	wikileaks founder assange
0	albright want attend nato seminar dc around feb like possible
0	reach
0	fyi michelleti call confirm yet offer time hear back hopefully willknow morning
0	suggest dan follow edit thegrafs page sure font showingup different get idea canimprove language want give senseof think still miss late draft
0	dear bill tomi sorry hill today long plan grateful thedepartment appreciate leadership everyday nursing crack head cheer remain calm carry happy holiday hu department statecase doc date state dept produce house select benghazi comm subject agreement sensitive information redaction foia waiver state scb
0	find course burg try herd cat see back dc
0	reed two book sed nothing mtp arrive soon
0	pis note calendar thx
0	dear hillarythanks encouragement nato honor ask work fascinate challenge leave slovenia tomorrow second seminar go prague celebrate velvet revolution touch iget back another update ministerial figure meetall bestmadeleinesent use blackberry
0	print hrc late version doha speech sure others edits want copy
0	fyi israeli spin storm start plane brief way
0	burn go happen commit herwork doc like lyn heard rumor acompetitive hospital etc work hrc event hospital
0	fyi thanks sign book hope get royalty mongolia also think would like tosee attached letter president clinton
0	tutu gail collins work address call grid send update grid huma withinthe hour gail collins book desk return
0	set turkish fm call tomorrow
0	yes stephen dc day work ops call think need push angolan minute fine move forward adjust
0	trust instinct go small group mtg bus kurt jeff much moreoptimistic sense overall direction mtg right base conversation bus themtg would ask tough question put
0	go happy go record anytime anywhere behalf let know
0	first heard
0	call service pi call soon negotiate middle eastpresidential statement come disagreement lavrovirussians reach susan tell consult washingtonand call back pls call get car jake also aware
0	ok need call sheet update burn steinberg
0	yes problem take vaniii meet la guardia
0	let pj respond honestly know execute
0	dan
0	office official travel tuesday november th need immediate assistance please call
0	suppose
0	call around pm
0	congratulate weekly meeting monday securing agreement
0	sound good
0	fax broken huma come print try conference jake discus thx
0	little change substance language still work though
0	say repeatedly want see palau shortchang know whether assessment accurateso pls get update recommendation take care
0	go skip yale today assume ok

0	thx home hour check
0	ask special fax draft chappaqua get shortly hopefully start feedback welcome course
0	tprrihh corn receive message reason get memcon call work getpoints together followup call weekend
0	french docx final communique portuguese docx finalcommuniquecreole revision docxfyi
0	well deserve rock star
0	wait send clean version
0	get draft joint communique carefully haggle language detention booktonight also get propose quartet statement prox talk happy mother day
0	reach thx
0	cloture invoke lael brainard nomination afternoon miguel rodriguezdeputy assistant secretary senate affairsbureau legislative affairsu department state
0	time since pc drop also leave friday later thurs friam
0	surprising assessment embassy
0	message deliver recipient
0	sure srap forward hope trip go well jm
0	story richard call talk statement draft review
0	still get confirm today
0	meeting first stop tell oscar pack commencement robe unless want use barnards would take back dc otherwise
0	update yesterday still wait today update dan work language numberof point get late either late tonight tomorrow morning base discussion fardraft statementdiscussionanalysisfor merit
0	yes already work
0	think several folk toss wrench email unclassifi
0	rahms assistant ask email address want give
0	hiattach new draft try pack change addition request yesterday wellas cheryl lissa suggest edits use metaphor rather simile season stage bit like decorate achristmas tree try hang ornament branch bend much whole tree stay upright want get first circulate others send oscar helpful termsof get hard copy dan
0	hope chance read sent yesterdayand great pic time today
0	yes send within hour
0	meeting quinn mean much perfect capstone year see shanghai
0	would happy see tomorrow immediately follow small group meet two minor addition phone call columbian president uribe request confirm jack lewwould like see around pm talk trade leave trip work
0	hear great diving
0	stop one home heading
0	also pls pas saltthx
0	yes lona list
0	letter good go hrc
0	yes copy
0	see original email pa
0	thanks get hope think carefully vp follow item
0	meant thx
0	little thing go long way thanks touch
0	dear tom december pmcould schedule call several matter like discuss weekend hop schedule call free tonightafter tomorrow btw sunday btw ham pm time work way still laugh yurt hillary
0	remind discus

0	wexler come pm menendez pm ops connect
0	like send letter memorial service call family
0	thx much
0	blair finish bn want talk available
0	dan feldmanimportance highi involve preparation holbrooke memorial service kennedy center jan need get guidance decision role reachable ops oron cell although pocket thisafternoon evening regard strobestrobe talbottpresident brookings institution massachusetts avenue nwwashington dc
0	well weekend plane little plane call mario see
0	thanks see meantime happy trail send note along way
0	readout mitchell meet ashton recommendation toi send point huma david follow russian envoy heard lavrov david feel comfortable youb declassify
0	pls sure letter wife friend ronnie add call list
0	let know think
0	margo amisabelle enjoy time glad come
0	put guy back front foot come look forward discuss
0	madam secretaryi receive copy draft night note colombia panama ftas review see anynotes guidance would like thanksmikemichael fuchsu department state cell
0	assume cdm already share
0	call sheet way steinberg clear
0	clear confirm thank sorry fire drill
0	tried emailing sure go missed shaun talk co short ago say still detail work monday put full stop though thing look good tomorrow day llreconvene monday bang last detail hopefully announce touch person morning give report also check decian
0	sbwhoeopsunday october pmre boehner poll sidgood tell shaunsent via droid verizon wirelessoriginal message
0	pls see note rich
0	free quick dinner lunch next week
0	try call troop camp leguene deploy afghanistan want picture etc
0	westewelle call holbrooke say need talk
0	okay window
0	senate may move start tonight fail cloture vote fy budget tax issue
0	call several item
0	ok also talk lavrov call
0	pi go today tell sorry want go ahead today call emailto tell happen
1	good day warm heart offer friendship greeting hope letter meet good time however sincerely seek confidence transaction propose free mind person integrity get contact result discreet search associate country know mail must come surprise meet maintain theory business person contact hence mail particularly interested deal expect reliable helpful name mr oliver yengeni south african citizen young brother mr tony yengeni brother mr tony chairman parliament south africa former chief whip rule political party anc brother mr yengeni result trust confidence mandate search reliable trustworthy foreign partner assist receive fund cash total twenty seven million five hundred thousand dollar personal company reliable foreign bank account safe keep period time since family bank account within outside country frozen authority would refer three website information family really require assistance iii still want let know money custody private security finance company europe believe trustworthy person take advantage money get total sum share follow assistance offer family use reimbursement expense incurr process transaction risk involve put modality place see success project must call meif interested assist urgently reply via email address please endeavour keep transaction confidential glad come forward help thank advance anticipate assistance enable achieve goal god bless oliver yengeni nb agree term kindly matter urgency send email due sensitive position south african government would want call phone send fax correspondence must email

1	hello dear get contact internet search browse want establish business relationship mr momohh ngogbee branch manager national bank dubai ummal quwain branch dubai united arab emirate marry fourchildren write letter ask support co operation tocarry business opportunity department june oil consultant contractor dubai petroleumcorporation mr david ameeraly make numbered time fix deposit fortwelve calendar month value sixteen million five hundred thousand dollar branch upon maturity send routine notification forward address get reply month send reminder finally discover hiscontract employer dubai petroleum corporation mr davidameeraly die plane crash october egyptianboe flight passenger board confirmit via websitebelow investigation find die without make willand attempt trace next kin fruitless therefore make investigation discover mr davidameeraly declare next kin relation hisofficial document include bank deposit paperwork bank sum interest roll principal sum end year one ever come forward claim accord united arab emirates law expiration ten year money revert ownership united arab emirates government nobody apply claim fund proposal like stand next kin mrdavid ameeraly fruit old man labour getinto hand corrupt government official simple iwill like provide immediately full name address thati forward contact bank attorney prepare thenecessary document affidavit put place thenext kin bank account part world provide willthen facilitate transfer money thebeneficiaryext kin money pay account share ratio set aside expense incur business risk paperwork transaction do attorney myposition branch manager guarantee successful execution ofthis transaction interested please reply immediately upon response shall provide detail relevant document help understand transaction please observe utmost confidentiality rest assure thistransaction would profitable shallrequire assistance invest share country please upon receipt mail message send followingbelow enable start process full name house address phone number fax thank god bless sincerely mr momohh ngogbee bf bd
1	dr jaes morgan principal auditor nigeria national petroleum corporation nnpc falomo office complexikoyi lagos sir guess letter would embarrass since ihave previous correspondence theforeign trade office nigeria chamber ofcommerce andindustry give company detail requesthowever center mutual collaboration yourunflinch support confidentially set ajoint venture outfit country hope would regret approach thismatter present work chairman taskforce review previous presentcontract award various ministry thecountry appointment office thepresidency federal republic nigeria sincethe appointment take effect november thetask force jointly discover someirregularities comprise invoice contractvalues meantime discover ninehundred eighty million united state dollarsus respective position status anopportunity enrich family amatter fact timely opportunityconsider economic situation country wehave agree declare nine hundr fifty sixmillion united state dollar tothe government balance twenty four million united statesdollars remit yourprivate company account hope establish joint venture business youin country money accept beus financing cost equity otherexpenses take joint business wehave set machinery enable secure theeffective remittance fund account ifonly work however noteworthythat business extremely sensitive must bekept confidential furthermore money duefor compensation effort andassistance incidental expense incurredon course transfer fund therest go joint venture project please notethat remittance would effective workingdays receipt bank accountdetails follow bank name address account number beneficiary name address private telephone fax number easycommunication confidentiality thistransaction however endeavour reply capacitydoes meet proposal canfurther negotiation good regard dr james morgan
1	second mail dear sir madam mr azzam mahmud chairman tender committee one lead oil company committee principally concerned payment contract award date order priority regard capital project corporation positive convince would provide solution money transfer deal value million thirteen million united state dollar subsequently joint business venture need bank account country offshore account would like invest money manufacturing product household product electronic good apparel textile real estate property telecommunication country idea investment would welcome course duty value project inspector go liquefied natural gas lng project invoice value job do foreign contractor tune million
1	sir compliment day life general withyou course humble wish solicit andcrave indulgence make project request fora joint business transaction hope notcome surprise hence plead foryour pardon dr sam jordan manager headquarterfirst bank nigeria plc lagos urgent andconfidentialbusiness proposal june italian oilconsultant contractor via national petroleumcooperation nnpc mr paul bush make numbered time fix deposit twelve calenderer month value atus branch maturity send routine notification hisforward address get reply month wesend reminder finally discover hiscontract employee nigeria national petroleumcorporation mr paul bush die anautomobile accident investigation wasclear die without make allattempts australian trace next ofkin fruitless therefore make investigation discoveredthat mr paul bush declare next kin orrelations official document include hisdeposit document bank total sumus still bank dormantacct one ever come forward claim accordingto nigerian banking law five year moneywill revert ownership nigeriangovernment account owner certify deathand nobody come forward claim situation proposal amlook foreigner stand thebeneficiaryext kin simple haveto immediately send detail bankaccount anywhere world arrange theproper money transfer document money betransferr account share theration remain set aside expense incur withhere risk involve use position andconnection bank

	documentwork transaction shall employ servicesof attorney draft obtain allnecessary document letter administration inyour fovour transfer transaction riskfree guarantee interested please replyimmediately confidentiality rest assure thatthis transaction could profitable ofus please expose deal notinterested take outof seat please expense involve thisbusiness phone fax number bank particular await urgent reply good regard dr sam jordan
1	kindly accept apology send mail true god fear person want trust help condition believe highly respect personality consider fact source profile human resource profile database country internet though know extent familiar event fragile political situation liberia form consistent headline cnn bbc news bulletin name mrs suzana nuhan vaye liberia country west africa late husband issac nuhan vaye deputy minister public work liberia husband falsely accuse plot remove president liberia charles taylor office without trial charles taylor kill verify international newspaper post web site husband kill move sum million diplomatic mean deposit security company abroad money meant importation agricultural machinery
1	sir serve officer custom exercise country duties international airport confiscate box come country china open discovered genuine dollar note well pack lace material honest dedicate officer hand seize boxes government investigation discover box bring retired general disgruntle politician truncate political dispensation surprise theses box hand money use area election recently swear ceremony president come across another set six box contain item officer know process apply take two box contain usd sixty million dollar take country serve officer allow operate foreign account see involved business kind therefore seek assistance collect money invest time retire offer acceptable compensate certain percentage work party agreement drawn protect party kindly assist duly reward inquiry please send mail mail box get reply provid telphone number please note attend request kindly destroy document joepardise position work reply alternavite thanks cooperation mr oki james
1	three thousand dollar account attn feel quite safe deal important business go remarkable profile internet though medium internet greatly abuse choose reach still remain fast medium communication however correspondence unofficial private treat first like assure transaction risk trouble free party name mr tony hill work operational manager account management section charge credit foreign bill one prime bank south africa routine inspection discover dormant domiciliary account balance forty eight million two hundr twenty three thousand dollar discreet investigation also discover account holder die long ago family fatal auto accident leave beneficiary account person know account close contact manager bank partner willing approve transfer reliable trustworthy foreigner foreign bank account former operator foreigner australia particular certainly sure writen oral attach account beneficiary nobody come claim money foreigner claim money legal claim account holder provide interest therefore need cooperation transaction difference country origins hindrance success transaction money remain useless dormant account transfer fund foreign account provide necessary information document need order claim money need cooperation foreigner provide safe bank account information transfer arrangement ensure smooth transfer fund account nominate already place also provide direct phone line easy communication wish utmost confidentiality handle transaction conclussion transaction share total transfer sum partner set aside expense party might incure process transfer fund transfer account two part first transfer usd million account fly meet successful transfer share accord agree percentage remain balance usd million transfer account successful share first transfer without disappointment side look forward reply mail call fax want call dial way use call country put south african area code satellite phone bourght transaction security reason local land line secure let hear good regard mr tony hill search browse smarter faster download msn search toolbar today free
1	instruct contact abacha family family former nigerian military dictator head state general sani abacha die th june office assistance matter state consult service retain late general wife currently house arrest result family recent confrontation present government federal republic nigeria client family account switzerland amsterdam country recently freeze bead nigerian government get hold late general sani abachas money recently general first son arrest murder charge commit also attorney see attach news paper publication however late general money hide west african country care security finance company tune cash nigeria government know please keep confidential fund move country via diplomatic mean late general reign power family would want immediately travel europe fund lodge contact security courier company case travel europe work fast way take possession money filter profitable business venture avoid nigeria government cease money effort expect take money abachas family currently allow assess account outside country relay heavily trust friend money late ordeal information visit weblink desperately contacting time essence inform one desposit security courier company amsterdam return back end year clear possess
1	proposal meet intrerest decide give anegotiable percentage entire sum hop besatisfactory please send along response follow confidential private tel fax number contact name address thank much anticipate urgent response respectfully dr kelvin promchay
1	task accomplish want anything jeopardize lastwish due fact want relative family member stand way last wish love jennifer wilsonhello name mr jennifer wilson die woman decide donate year old diagnosed cancer year ago immediately death husband leave everything work touched god

	donate inherit late husband good work god rather allow relative use husband hard earn fund ungodly ask god forgive sin believe merciful god go operation pray survive operation decide donate sum one million five hundred thousand dollar good work lord help motherless less privilege also assistance widow moment take telephone call due fact relatives around restrict doctor take telephone call deserve thee rest get presently inform lawyer decision willing fund wish good may good lord bless abundantly please use fund well always extend good work others kindly contact lawyer email address interested carry task arrange release fund lawyer name brown walter
1	usiness fact decease man foreigner authorize law guide bank citizen country ke claim fund request foreigner come business necessary facilitate release transfer fund smoothly reliable bank account survive rel ation decease br br really sure integrity tru stworthy confidentiality accept cheat disappoint afte business reply telephone fax number bank acco unt detail wish receive fund br br upon receipt mention information indicate interest business wi send fax mail letter application type send back bank enable start transaction br br fail bring notice business risk free negative implication entertain fear odalities smooth easy transfer fund finalize transaction complete within bank work day immediatel start business br br wait urgent response please kindly delete interested br regard br br nbsp mr nbsp
1	audit find government official set company andaward contract grossly invoiced variousministries also identify lot inflate contract fund arepresently deposit apex bank accra however virtue ofour positionas civil servant member panel acquire money inour name therefore delegate matter trust mycolleagues panel look overseas partner whoseaccount would transfer total sum usd thirty
1	dear sir name mohamm hamid iraq cousin top officer former government sadam hussein begin war give seventy five million united state dollar keep manage move box contain money iraq diplomatic immunity security company london box deposit family treasure security reason war want move money country safety reason rest assure transaction risk free person aware money compensate total sum assist expect urgent reply via faithfully mohamm
1	already know time great essence transaction kindly confirm telephone fax number mr mike obodoh bear mind project conclude within five day prompt response kind personal regard dr ben mboye edb bbb fdccd
1	challenge security finance ltdlagos nigeriaattention siri mr isa muhame director operationschallenge security ltd lagos nigeria innigeria firm security company high reputewith year outstanding service person ofafrica resolve contact thismedium base business proposal ofmutual benefit get particular consultant companybase research atrustworthy establishedperson understand investment ethic enteringinto life time profitable joint partnershipinvestment also co operation confidence andtrust willkeep content secret anddivulg third party explicit straight point timeearly reputable client depositedaconsignment company vault safekeep since client fail comeforward claim consignment hasaccumulat aconsiderable amount money indemurrage consequently bide contact thisclient redeem demurrage consignmenthad accumulate discovered client theformer president federal republic zaire whodi illness de throw sameyear consignment entrust care sincethe death client president mobutou seseseko none benefactor come forward claim theconsignment mean non hisrelatives aid knowledge thisconsignment hence curiosity decide secretly open thetwo box client deposit vault andto surprise discover two box thatwere register treasurer client actuallycontain considerable amount money unitedstates dollar amount milliondollar since development nursingplans secretly also find enquiries andthe foreign medium late client siphon lotof money country office ashead state conviction consignmentin vault part money clientsiphon dead race tothis money care solicit nobleassistance assist transfer money outof nigeria country immediate investmentwith assistance also decide willgenerously title total amount upon receiptof reply confirm yourwillingness assist transaction willimmediately arrange transfer right ofownership consignment name tofacilitate easy clearance transfer thecomplete fund yourcountry nothing worry thereto assist anyway necessary properdocumentation transaction risk free please maintainabsolute confidentiality matter please reply email address yourtelephone fax call thanks faithfully mr isa muhame get tiscali broadband
1	mr henry kaborethe chief auditor inchargeforeign remittance unit african development bank adb ouagadougou burkina faso dear partner presume well family please let surprise message get contact information fromthe international directory week ago decide contact magintude lucrative transaction present future survival life moreover lay solemn trust decide disclose successful confidential transaction chief auditor incharge foreign remittance unit bank decide contact financial transaction worth sum nineteen million three hundred thousand united state america present future success abandon fund belong one bank foreign customer die along entire family plane crash disaster since year ago meanwhile fortune come across decease file arrange old abandon customer file sign submit entire bank management official documentation audit year inform clearly state foreign banking rule regulation sign lawfully fund remain unclaimed till period year start date beneficiary die money transfer treasury unclaimed fund however authorize rule guide bank citizen burkina faso make claim fund unless foreigner matter country

	different decease request foreigner necessary apply claim transfer fund smoothly reliable bank account next kin decease fund transfer account thirty five percent respect assistance transfer fund account provision bank account bank remit fund fifty five percent pioneer business rest ten percent share respectable organisation center charity motherless baby home helpless disabled person around world really sure trustworthy accountability confidentiality transaction contact accept change mind cheat disappoint fund transfer account reply assurance telephone fax number necessary facilitate easy communication soon reply let know next procedure follow order finalize transaction immediately expect urgent communication sincerely mr henry kabore search find check new msn search
1	great honour write several hesitation want say much name bakri definitely tell get know definitely get reply diagnosed ideopathic pulmonary fibrosis terminal disease chance survive write cooperation fufill last wish lay death bed wish distributing last wealth keep diplomatic box security company diploamtic box contains huge sum money would reveal later want stand behalf collect box help distribution wealth less priviledged expect response perform task several person die suffer world abd feel widow mite change thing little help less priviledged fund count good wishesk bakri
1	desk mr muham hassanebill exchange managerafrican development bank adb ouagadou burkina fasodear friend message might meet utmost surprise however urgent need foreign partner make contact transaction banker profession burkina faso west africa currently hold post bill exchange manager bank african development bank adb opportunity transfering leave fund million one bank client mr andreas schranner die along entire family july plane crash living nobody behind claim leave fund bank confirm genuiness decease death click web site invite business deal money share ratio agree business proposal detail transfer forward soon receive return mail great day faithfully muham hassanebill exchange managerafrican development bank adb express instantly msn messenger download today free
1	zhang bank china shenzhenchina dear friend zhang bank manager bank china shenzhen branch china urgent confidential business proposition june consultant contractor chinese solid mineral corporation make numbered time fix deposit twelve calendar month value twenty one million dollar branch upon maturity send routine notification forward address get reply month send reminder finally discover contract employer south korea solid mineral corporation die along wife plane crash investigation find die without make attempt trace next kin fruitless therefore make investigation discover declare next kin relation official document include bank deposit paperwork bank sum still sit bank interest roll principal sum end year one ever come forward claim accord law china expiration eight year money revert ownership south korea government nobody apply claim fund consequently proposal like foreigner assist receive money every machinery set enable receiver money via finance firm base overseas need urgent assistance money get hand corrupt government official simple require send complete name contact address telephone fax number able apply necessary document enable receive money via finance firm overseas name receive money share ratio note project risk free consider position credit officer bank guarantee successful execution transaction facility obtain relevant document enable lift money bank overseas interested please reply immediately upon response provide detail relevant document help understand transaction also let contact detail finance firm country locate overseas please send confidential telephone fax number fast communication need speak personally observe utmost confidentiality rest assure transaction would profitable please reply secure email await urgent reply thanks regard zhang
1	receip mail indicate interest want assure risk attach transaction also provide private telephone fax number easy communication george lawson
1	aneousexpenses bear mind remain fund thesurvival family nevertheless reason intreste din proposal kindly inform start look anothe rreliable contactthanks wait immediate responseplease view website see myfamily name mess truly dr mr mariam abacha
1	dear friend know letter may beat imagination previous correspondence iam contact see confide introduce self mr ahm ibrahim tarlor son late former angolian diamond merchant like transaction one production father late diamond merchant make sum usd cash want invest money country assistance money question security company since father death moment confuse handle huge amount money also one confide ouagadougou burkina faso may fore use medium torequest dispose enough help keep huge amount money country place choice intend invest money profitable business venture give total money assist confirmation willingness assist reply telephone fax number easy communication good regard mr tanko usman express instantly msn messenger download today free
1	th may kind attention transfer fund investment cum joint venture discover account late customer office credit office rof bank london interest transfer fu ndworth million pound account country collaborator please indicate interest immediately proce respond soon possible follow full name phone number contact address occupation position age account detail use zero balance account dor mant fund transfer nominated bank account wil lcome country share get touch meon mobile number clarification detail

1	auditorhead departmentbank scotlandunit kingdom attention attention attention confidential business dear friendi want transfer fifteen million pound sterling bank herein scotland courage look reliable honest person whowill capable important transaction believe willnever let either future dr frank jim auditor compute staff bank inscotland united kingdom account open bank andsince nobody operate account go throughsome old file record discover remitt thismoney urgently forfeit nothing money come owner account mr john hughes foreigner manager ofpetrol chemical service london chemical engineer professionand die since since nobody know accountor anything concern account beneficiary myinvestigation prove well company know anythingabout account amount involve poundssterlingsi want transfer money safe foreign account abroad tknow foreigner contact foreigner thismoney approve local bank scotland onlybe approve foreign account money pound sterlings andthe former owner account mr john hughes foreignertoo well get email address form magazine advert know message come surprise know ourself sure real genuine business believein god never let investment transfer approve payment schedule allocatedoverseas offshore pay delegate final clearance andsign payment release form beneficiary want see atthe oversea pay clearance office face face sign originalbind agreement bind together receive money intoa foreign account account choice fund beremitt contact need involve foreigner aforeign account real beneficiary need co operation makethis work fine management ready approve payment toany foreigner correct information account willgive sure capability handle amount strictconfidence trust accord instruction advice mutual benefit idon want make mistake need strong assurance trust ishall destroy document concern transaction immediately wereceiv money leave trace place use position influence staff effect legalapprovals onward transfer money account appropriateclearance foreign payment department assurance moneywill intact pend physical arrival country share
1	dear christ heavy heart reach regard death late client barrister robert beaud legal adviser late mr mike carol hall wealthy couple live united kingdom recent death th december tsunami disaster last year couple serve god dedicate christian dedicated life god child till die acquire lot land property like land stock bond house property etc legal adviser death husband mr mike carol hall instruct write child dedicate wealth god accord asset give ministry work god legal adviser document asset deposit security company care give authority give fund ministry work god instruct mr mike hall death matter fact total amount money contain two trunk box want management security company know content consignment therefore register content consignment gold bar security company believe deposited gold bar born christian reading bible lawful right sight god give fund choose ministry purpose god work instruct owner death fast prayer today ask god make choice direct honest christian choose ministry deserve fund grace come across address internet browse christian site appeal fund use wisely thing glorify name god notify security company deposit consignment contain fund move consignment abroad security company await authority move consignment shore country dear child zion know use fund honestly wisely thing glorify god name contact mention information via prompt response highly appreciated christ barrister robert beaud
1	attn dr zingiwa micheal director south african refugee camp member salvation army unit six base joberg south africa oblerg intimate new development widow sudan recently transfer camp lose husband three child recent trouble darfur sudan tell come south africa huge sum money tune fourty million dollar million concell consignment deposited private security company pretoria proper keep safety want assist get reliable foreign friend assist transfer huge sum foreign bank account abroad investment purpose tell money belongs uncle head sudanese army kill also upheval remain greatful get contact pleased grant opportunity work transaction intoregat widow collect data prove correct sincere unfortunate exhaust true picture proposal write secret nature therefore opinion well inform need arrange person person meeting near future immediately indicate willingness assist assure trustfully unitedly execute transaction without risk cooperation benefit hope face face meeting surely enhance relationship give opportun ity know exactly percentages money finally transfer service charge also hope consider person position reply favourably personal mail address thanks sincerely dr zingiwa micheal yahoo tire spam yahoo mail good spam protection around
1	mr joseph cabutti silver crescent enugu nigeria compliment dayi joseph cabutti consult auditor one prime bank herein nigeria search trustworthy individual firm lead toyou come across contact look worthwhile investmentsor project affiliate write letter toask support co operation carry transaction propositionan industrialist foreigner customer client bank whodi th july concorde plane crash flight af withthe whole passenger board course audit discoveredan account open bank great late ndustrialist whodi without write oral attach account since hisdeath investigate kin interest see get anyrelative late industrialist prove abortive onehas come claim fund person know accountor anything concern account beneficiary death ceo ofhis company work client bank balance usd thirty six million united state dollar bank expectsa next kin claim beneficiary valuable effort make toget touch relative success ofthe perceived possibility able locate next kinand beneficiary management influence chairmanand member board director bank make arrangementfor fund declare unclaimed lose federalgovernment subsequently donate trust fund arm andammunition enhance course war africa order toavert negative development seek permission youstand next kin late beneficiary fund usd wouldbe release pay

	account beneficiary next kin wewish start first transfer ten million uponsuccessful transaction without disappointment side shallre apply transfer remain balance account secretly discuss matter close confidant attorney whomi must involve order assist produce legal back document whichwould substantiate claim also enable smooth andsuccessful transfer fund foreign bank account arego nominate note secure probate orderof mandamus locate decease beneficiary areassuring business risk free involvement notentertain fear modality fund transfer finalizedwithin banking day apply bank beneficiaryof fund decease conclusion transfer ofthe fund give total transfer sum charity africa country remain set aside settle expense party might incure thetransfer process proposal acceptable make undue advantage thetrust bestow kindly get immediately please furnish private telephone fax number easy andsafe communication respectfully joseph cabutti
1	hurricane katrina hurricane wilma south asia earthquake toother charity organization around world effort andtime talk phone due health situation amuse lap top computer communicate respond tothis mail interested carry assignment mybehalf reply mail address interested god mr simon robson
1	requestin order transfer fifteen million pound sterling bank london courage look reliable honest person capable important transaction believe never let either future mr jim harry auditor head compute department bank scotland united kingdom account open bank since nobody operate account go old file record discover remitt money urgentely forfeit nothing money come owner account mr lewis mowan foreigner manager petrol chemical service london chemical engineer proffession die since since nobody know account anything concern account beneficiary investigation prove well company know anything account amount involve pound sterling contact need involve foreigner foreign account real beneficiary finally conclusion business give total ammount expense party might incur process transaction urgent respons need thanks mr jim harry
1	office auditor general citibank kensington high stlondon nsdear friend dr robin quest chief auditor general citibank kensington high st london ns urgent confidential business proposition may iraqi foreign oil consultant contractor british petroluem corporation mr dara baraz make numbered time fix deposit calendar month value thirty two million two hundred thousand dollar bank upon maturity send routine notification forward address get reply month send reminder finally discover contract employer british petroleum corporation mr dara baraz die result torture hand sadam hussein one trip country iraq investigation find die without make attempt trace next kin fruitless therefore make investigation discover mr dara baraz declare kin relation official document include bank deposit paperwork bank sum float unclaimed since bank effort get relative hit stone accord british law expiration six year money revert ownership british government nobody apply claim fund six year end september consequently proposal want seek consent foreigner stand owner money next kin decease bank transfer money desi gnat account document prove enable get fund carefully work secure probate order mandamus locate decease beneficiary assure business risk free involvement share stay rest investment purpose leave london end year share fund base accord agreement within soon receive acknowledgement receipt message acceptance mutual business proposal furnish necessary modality disbursement ratio suit party without conflict proposal acceptable take undue advantage trust bestow please appreciate fact business internet risk endeavor send confidential telephone fax number inyour reply business transaction regard dr robin questauditor citibank london
1	mr maria socorrocredit account officerhead office equitable pci bank twr mkt avenue cor dela costa strphilippines good day let start introduce mrs maria socorro credit account officer equitable pci bank write letter base late development bank like bring personal edification write letter much joy excitement even though heart go powerfuland distinguish gentleman fortunate work extremely privileged know numerous year top official charge client account equitable pci bank inside philippines client go horrendous divorce united state america verge lose estate vicious diabolical wife result alarm predicament client come brilliant idea transfer fund ten million two hundred thousand dollar fixed deposit account bank alias two know confidentiality matter necessary protection due untimely death early fund sit account ever since continue perpetually unless something come locate agency help seek person email client declare next kin official paper include paperwork bank deposit backdrop suggestion would like foreigner stand next kin client able receive fund want know everything plan come successful contact attorney prepare necessary document back next kin client require stage provide full
1	greet name lord jesus christ mr prisca konan widow late martin konan year old new christian convert suffer long time cancer breast indication condition really deteriorate quite obvious live six month accord doctor cancer stage get bad stage late husband die air crash return business trip europe te ivoire period marriage produce child emy late husband wealthy death inherit business wealth doctor advise may live six month decide divide part wealth contribute development church africa america asia europe pray willing donate sum md es dollar less privileged please want note fund lie security company upon instruction attorney presently africa distribute relief material bomb last sierra leone file application transfer

	money name lastly honestly pray money transfer sure say purpose come find wealth acquisition without christ vanity may grace lord jesus love god fellowship god family ei await urgent reply christ emrs prisca konan
1	hello son immediate past minister anafrican country remove office becauseof aledg million memberof nationl identity card scheme thatcountry go brought thecountry independent corrupt practice otherrelat offence commission icpc ofhis personal account within outside thecountry frozen use medium solicit yourassistnce trnsferr huge amount moneymy father deposit security firm europe asof date fund yet discorver base onthis assist transfer fund toyour personal account shallre invest fund legitimate business thatit trace father willing toassist initial sum afurther whatever investment go need spell importance theneed secrecy consider volatile nature ofthe matter party involve assume thatmost phone fax line tappedso willing asist list yourphone fax number contact isimportant tallk themodalities transfer time great essence matter donot hear within next three day willtake interested solicitfor new partner wait hear oladimeji afolabi
1	computer optimization installation installation monaxturbine turn around maintenance aim invoice payment divertthe excess amount discrete account abroad fund floatingin suspense account central bank nigeria cbn fund colleague decide transfer youraccount since civil servant allow operate ownforeignaccount money share follow transfer account owner colleague set bothlocal international expense thatwould incurred course ofthis transaction able claim fund purport company bethe original contractor beneficiary fund procedures forinternational transfer shall strictly follow work outall modality swift riskfree transfer proposal satisfy please contact via mail fax number follow important information bank name address account nameaccount number personal phone fax number easy communication transaction last working day time submit require information modality concern transaction work completely risk free please inform subject classify sensitive therefore treat transaction utmost confidentiality urgency faithfully dr martin awele broadband unbeatable
1	extension pipeline network within nigeria crude oil stream product distribution subsequent evacuation million contract turn around maintenance tam various refinery country million construction storage tank petroleum product depots million original value contract deliberately invoice sum million american dollar approve ready transfer company actually execute contract fully pay project officially commission consequently colleague willing transfer total amount account subsequent disbursement since civil servant prohibit code conduct bureau civil servant law open operate foreign account name needless say trust pose juncture enormous return agree offer transfer sum shall set aside accidental expense internal external party course transfer mandate draw investment plan control since bring share back nigeria interested invest real estate country must however note
1	quote sum profile fund name enable european bank transfer sum decide use sum relocate american continent never connect mikhail khodorkovsky conglomerate soon confirm readiness conclude transaction willprovide detail thank much regardsnepyyvoda petro mr
1	dear one christ good day sorry take time disturb mr susan thompson kuwait marry late mr eric thompson work kuwait embassy ivory coast twenty six year die year brief illness last five day marry eighteen year duaghter lilian later die motor accident untimely death husband bear christian since death decide remarry get child outside matrimonial home bible late husband alive deposit sum fourmillion eight hundred thousand united state dollar general trust account prime bank abidjan cote ivoire presently money still bank recently follow ill health doctor tell may last next eight month due cancer problem one disturbs stroke sickness know condition decide donate fund christain organization church individual beleiverthat utilize money way go instruct herein accord desire latehusband death want fund use christain activity like orphanage christain school church propagate word god endeavor house god maintain bible make understand bless hand giveth take decision child inherit money husband relative christian want husband effort use unbeliever want situation money use ungodly way take decision afraid death hence know go know go bosom lord exodus say lord fight case shall hold peace need telephone communication regard health hence presence husband relatives around always want know development god thing possible soon receive reply shall give contact bank abidjan also issue document prove present beneficiary fund want member church always pray lord shephard happiness live life worthy christian whoever want serve lord must serve spirit truth please always prayerful life delay reply give room source another person purpose please assure act accordingly state herein hop receive reply remain blessed lord christ mr susan thompson protect pc yen security campaign
1	attentiondear delight write letter hop und erstandmy predicament answer back without hesitation mr mariam abacha wife late nigerian head state general sani abacha get yourcontact business inquires chamber commerce wasmak contact honest foreigner help save life mychildren hence country frustrate since death husband possession thirty five million dollar want invest country clarification understandin money truck box deposit security companyindisguise family treasure diamond gold since th may ill get wealth rather will late husb acceptance render assistance instruct thesecurity company effect change ownership fund yourname bona fide owner next kin enable retrieve fun dsince box move country family name

	important forward full name privatetelephone fax number occupation enable send relevant docum entspertain deposit fund bear mind transa ctiondemands absolute confidentiality property asset bankaccounts local international bearing abacha family name confiscate already elect civilian president nigeria call president olusegun obasanjo hence want expose dthis aviod lose money knowledge fund thirty five million dollar give assist secure fund set aside expense might incur process
1	sum arise deliberated invoicing contract awardedby ministry nigerian national petroleum corporation foreign firm inthe twinlight day last military regime contract completelyexecut commission contractor handle contracthas collect full final payment thus leave behind statedamount represent invoice sum safeguardingthis money conducive time transfer country ourpersonal use however code employment allow civil servant operate foreign account contract handledby foreign firm payment make dollar need foreignpartner present sub contractor fundwill transfer company personal account address wasmade available good friend work nigeria exportpromotion council assure company viability capabilityin business transaction assurance give courage link youup particular transaction hope mutual benefitto note nature business particularlyrelevant success transaction require willingnessto present subcontractor provide bank informationso fund transfer account share ratio assistance business share totalfund colleague take set aside expensesthat may incur course transaction willingto assist business send follow information name bank address account number beneficiary name private telephone fax number easy communication information enable put application payment approvalto concerned ministry final senate committee contractpayment go last fourteen work day start fromthe day receive information modality take offof transaction work action commenceimmediately hear wait reply fax thisfax number state faithfully dr john ejime
1	gnment also give detail address consignment deliver get back soon possible enble forward shipping document legally emback ceipt claim mean come country immediatel recieve consignment share reply private congrats remain blessed good regard faithfully dr clement asuzu span div div
1	task accomplish want anything jeopardize lastwish due fact want relative family member stand way last wish love jennifer wilsonprueba el nuevo correo terra seguro aacute pido fiable
1	name muthar bola ige eldest son slain attorney general chief justice nigeria chief bola ige get contact se arch reliable trustworthy partner business contact view fact great assistanc business proposal benefit reach private
1	belov sorry unannounced communication especially common method promise give reason due course nopparat sujaritpinij thailand work terry ford white farmer zimbabwe till sudden death march bos terry ford rich commercial successful farmer zimbabwe till land reform mugabe led government expose many white farmer brutality black war veteran one attack shot kill case familiar incident look international news medium like contact respect deposit fund tone million meant purchase farm tool accessory since death think way forward continuous stay fund bank government may trace mean total lose hence arrange withdrawal successfully withdraw fund deposited private security company family treasure well inform farming could business well need partner help settle fund account personal company account please rest assure shall like remain fund question nothing whatsoever zimbabwe longer urge feel free deal risk involve deal ready compensate effort total fund reimburse form expense side otherwise work partnership investment wait hear ever ready supply every detail well transaction regard nopparat afe aaa
1	assistance agree offer total sum investment country finally require maintain secrecy proposal god bless abundantly truly mrs aisha kabila family old zaire
1	client well know al qaida hizballah al jihad hamas abusayyaf group many terrorist group well knownsupplier market look forward expand clientagewith assistance internet hesitate contact via icq impatiently await order shadowcrew
1	dear one yuriy lagutin represent mr mikhail khordokovsky theformerc yukos oil company russia sensitive andconfidential brief top oligarch ask yourpartnership inre profile fund million give detail butinsummary thefunds come via bank menatep legitimate transaction youwill pay management fee interested please write back provide confidential telephone number fax number andemail address provide detail instruction pleasekeep confidential afford political problem finally pleasenote must conclude within two week please write backpromptly also suggest visit news sit internettobe good inform project back look forward regardsmr yuriy lagutin
1	dear friend wish begin way introduction kenneth mugaji first andonly survive son late david mugaji one foremost rich blackfarmers zimbabwe murder land dispute country death father give certificate depositehe use deposit sum million thirty million five hundredunited state dollar one private fudiciary company unite kingdom family valuable realize loom danger zimbabwe amount meant purchase new machinery chemicalsfor farm also establishment new farm land land dispute start president robert mugabe introduce new land reform particularly target rich white farmer black farmer father include result gruesome killing rich farmer mainly white andthe unlawful possession property mugabe war veteran disguise fight interest country isagainst background fled country family currently seek asylum decide seek foreign assistance law prohibit asylum seeker

	operate bank account involve financial transaction kind hence letter need trustworthiness able entrustthe say amount help investment opportunity family survival depend money virtually lose asset two option firstly choose accept totalamount assistance go partnership properinvestment money country shall discuss detail modality involve hear await reply medium implore tomaintain absolute secrecy require ensure safe hitch freetransaction shall furnish detail request truly kenneth mugaji
1	finally hereby advise indicate honorable office wit immediate effect person instruct mr john tsai come claim fund enable endorse final ayment approval order behalf call phone number clarification wait hear soonest rest assure professional service protect fund reply mail address confi dential reason john tsai take possession fund prof charles soludo executive governor central bank nigeria
1	treat urgent pls dear partner eattn fpls dear partner would like apply medium co operation secure opportunity investand joint business country substantial capital honourably intend invest country lucrative business venture advise execute thesaid venture mutual benefit eyour able co operation become business partner country create idea money invest cproperly manage type investment money transfer custody help assistance emeanwhile con indication willingness handle transaction sincerely protect interestsand upon acceptance proposal ci would furnish full detail information cprocedure camount involve mutually agree percentage interest share hold help secure release deposit invest money country proper management andcare eshall glad reserve respect opportunity cif desire cbut urge give matter immediate attention deserve eif proposal acceptable cplease make undue advantage trust bestow cand urgent call reply highly need cfor moredetailes information oral talk elook forward candid urgent call positive reply today mutual healthy business relationship ebest regard cand peacefully cool day eyours faithfully cmr edaniel william
1	matter also want guarantee partner myselfare good position make payment claim possibleprovid give strong assurance guarantee ourshare secure please remember treat matter veryconfidential matter comprehend form ofexposure still active government service remember onceagain time essence business wait anticipationof early response truly engr erick
1	kenya commercial bankcity centre box nairobi kenya sir madam may crave indulgence open business discussion informal letter sort pertinent business magnitude commence properly formal meeting enable party know fore knowledge nature business discus acquaint responsibility function party appropriate share accordingly dan wilson branch manager kenya commercial bank kenya urgent confidential business proposition june foreign oil consultant contractor kenya ministry energy mr clifford stone make numbered time fix deposit twelve calendar month value twenty one million five hundred thousand dollar branch upon maturity send routine notification forward address get reply month send reminder finally discover contract employer national petroleum corporation mr clifford stone die automobile accident investigation find die without make attempt trace next kin fruitless therefore make investigation discover mr clifford stone declare kin relation official document include bank deposit paperwork bank sum carefully fix bank safekeep one ever come forward claim accord south african law expiration five year money revert ownership government nobody apply claim fund consequently proposal like foreigner stand owner money fix deposit bank write public servant operate foreign account want present owner fund able claim help attorney simple like provide immediately telephone number full names address attorney prepare necessary document put place beneficiary fund money move share ratio paperwork transaction do attorney guarantee successful execution transaction interested please reply immediately via email upon response shall provide detail relevant document help understand transaction please observe utmost confidentiality rest assure transaction would profitable shall require assistance invest share real estate within country due nature confidentiality transaction communication via email fax mostly await urgent reply via private email thanks regard dan wilson branch manager kenya commercial bank febc ae bb
1	please interested assist carry full capacity transaction would require following information would enable make formal application various ministry parastatals release onward transfer money account full name company name address telephone fax number bank name address telephone fax number bank account number beneficiary name must signatory please note strong reliable connection central bank nigeria government parastatals hence assistance regard would problem conclusion transaction shall use contact withdraw document use course avoid trace whatsoever may ever arise near possible future might also interest know mere civil servant want miss opportunity hence want money transfer soon possible newly democratically elected government ever think make enquiry regard various activity past military government kindly contact soon possible whether interested deal whereby interested would give room scout another partner interested kindly contact via email telephone fax swing action time part wait anticipation full co operation faithfully dr goronyo baba send email address tel numberif interested verification actually receive mail

1	mr john mbuli native south africa base benin afriend late engr gert also present financial controller ofinternational financial bank cotonou ranch republic benin havean urgent confidential business proposition result ofwhat presently hand th december engr gert lebanese investor cotonou republic ofbenin among plane crash victim boe type air plane ofafrican transport union uta crash th december fidjrossebeach cotonou republic benin person die beirut bound flightvictims mostly lebanese national westerner others stillreport miss passenger survive please seek consent present next kin decease sothat side track account value million dollar fifteenmillion five hundred thousand dollar late engr gert could remittedto account next kin share theamount mutually agree percentage require honest co operation trust enable seethis transaction guarantee execute alegitimate arrangement protect breach law toenable discuss transaction contact kindlyprovide follow
1	mr ben anifiniacial trust banklagos nigeriacan invest twenty million five hundred thousand united state dollar control country amount transfer personal account soon receive reply indicate interest ability handle email back full detail let know full detail immediately indicate interest also necessary document regard fund forward immediately authentic risk free share ratio subject negotiationthank regard ben ani
1	account provide tranfer share pione er business rest use install joint compa ny country bear name particular whatever prof make company use help less privilege mothrless baby home charity organization world br br really sure trustworthy accountability confidentiality transaction contact agree change mind cheat disappoint fund get account besid entertain fear sure success insider bank ok br br please reply assurance include yo your private telephone fax number necessary effective easy commu nication transaction soon reply let know ext step follow order finalize transaction immediately br nbsp br expect urgent communication br sincerely br mr outaba jean br br br hr play free game earn ticket get cool pri zes join live search club href target new join live search club bo dy html bbbe ab df bed abf
1	thanks attention faithfully steve casey general manager le trans
1	yahoo co ukn upon interest forward thesecurity company contact immediately tell thisbusiness argue well withboth party respectfully joy williams wilson il tuo partner un bravo amante scoprilo con aiuto delle stelle ht tp
1	also send confidential phone number fax number expect transaction take ten working day shall discus share ratio modality get response please note transaction must top secret sincerely
1	sobe inform amount pay million rest bepaid latter expect urgent response email enable monitor payme nteffectively congratulation anxiously wait reply capt james williamsunit nation london ukunited nation monetary fund unmf
1	greeting uaehighly confidential ei decide contact email feel secure private nasser bin nasiri director procurement contract award ministry petroleum mineral resource bmpmr contact concern transfer million dollar fourty three million dollar sometime ago contract award corporation bmpmr japanese firm carry turn around maintenance rehabilitation work emirates petroleum product company eppco contract invoiced tune million forty three million united state dollar partner reach compromise transfer thefund foreign bank account accommodatethis fund modality set ewe disappoint last time senator late paul wellstone trust transaction nature tune million usd sat money apparent reason still hold money family kill plane crash last december still believethat good person still exist world willing try bearing mind god way fight confirm transaction mr clarence barnes uncle senator provide receive bank account email address cbarnes worldemail ecom phone enjoy collective money refuse give share believe shall meet doom senator afraid need total trust assurance must honest straight forward person make costly mistake senator go agreement ebas status senior government official top sensitive position trust stand claim money without assistance foreigner process require presence uae guarantee transaction execute legitimate arrangement protect breach law intend share money follow manner expense incur transfer contact immediately interested detail look forward fruitful business relationship urgent response good regard mr nasser bin nasiri
1	qin li wanglloyds tsb pacific limit hong kong branchtwo exchange square central hong kong give time staff lloyds tsb group plc hong kong attached private banking service amcontact co
1	company name apply payment backdate award contract favour company look forward th transaction solicit utmost confidentiality transaction bring detailed picture transaction ar please get touch either email address american internet fax number tel good regard dr paul bugatifreeserve anytime half price first month save mont
1	kojo adam account department ecobank cotonou attn friend mr kojo adam auditor accountingdepartment ecobank cotonou benin republic get yourvery good name mail address online classifiedpost net decide contact purely thepersonal conviction trust andconfidence co operate one another verylucrative business mutual benefit business ampropos respect sum eleven million one hundred eighty three thousand unitedstates dollar deposit dollar account bank belong mr moham salehibrahim die th july plane crash engineering contractor bank make several effort contact family moham saleh ibrahim relative prove abortive ashe identifiable kin man sum remain unclaimed ever since nobody come forward

	next kin management influence chairman member board director make arrangement fund declare unclaimable subsequently turn thereserve account bank background forego two colleague bank decide contact assistance partnership stand asthe next kin mr moham saleh ibrahim permission fund transfer private accountabroad beneficiary next kin mr moham saleh ibrahim proof claim necessary documentation becarefully work favour assure risk free involvement protection consequently find proposal acceptable wish assist expect urgent response uponreceipt shall discuss agree disbursement share ratio let therefore expect urgentresponse phone mail address please endeavour include private phone fax number also private mail available please keep proposal secret confidential thank good regard await urgent response faithfully adams faites un voeu et puis voila
1	message contain confidential information intend forspecifi addressee name addressee di sseminate distribute copy mail sender therefore accept liab ilityfor error omission content message ariseas result mail transmission please note reserve right monitor read emails send receive telecommunicat ion lawful business practice interception communication regulation
1	dr solomon azeez federal ministry petroluem resource lagos nigeria attn sir request assistance strictlyconfidential dr solomon azeez accountant ministry petroleum resource mpr member three man tender board charge contract review payment approval come know search reliable person handle confidential transaction involve transfer huge sum money foreign account may sound strange exercise patience read series contract execute consortium
1	fondazion di vittoriofoundation officer fondazion di vittorio italy foundazion di vittorio would like notify beenchosen board trustees one final recipient cashgrant donation personal educational businessdevelopment fondazione di vittorio establish themulti million group supported economic community westafrican state ecowas united nation organization uno theeuropean union eu conceive objective human growth educational community development celebrate th anniversary program vittorio foundation inconjunction economic community west african state ecowas united nation organization uno european union eu givingout yearly donation one million united statesdollars lucky recipient specific donation grant beaward lucky international recipient worldwide differentcategories personal business development enhancement oftheir educational plan least awarded fund usedby develop part environment ayearly program measure universal development strategy objective make notable change standard live ofpeople around universe america europe asia africa andall around vittorio foundation assure highestorganization standard courtesy united nation beliefthat achieve great positive change general welfare theuniverse program foundation doingeveryth possible get recipient notify donation note country country benefit fromthis donation beneficiary choose country allcontinents idea donation within ten year notable richness among many unusual person around world give many person opportunity get life stagewhere always want kindly note choose receive donation mean subsequent yearly donation get take time think spend donation wisely something thatwill last long time recipient eligible award thisdonation base random selection exercise internet websites millionsof supermarket cash invoice worldwide select among luckyrecipients receive award sum two million fivehundred thousand united state dollar charity donation aid thevittorio foundation ecowas eu uno accordance theenabl act parliament note beneficiary email addresseswere select randomly internet website shop scash invoice around area might purchase somethingfrom require fill form email executivesecretary qualification documentation process yourclaims contact office request data begiven donation pin number use collect thefunds please endeavor quote qualification number discussion
1	mr peter ndabakellcross streetmidrandjohannesburgsouth africa dear friend accept sincere apology mail meet personal ethic mr peter ndab staff account management section well know bank south africa one account hold balance twenty five million dollar dormant last operate past year investigation confirmation owner account japanese foreigner name engineer meiji iwatani die th january automobile accident since nobody do anything regard claim money family member aware existence either account fund information national immigration also state late engineer meiji iwatani single point entry south africa confidentially discuss issue bank official agree find reliable foreign partner deal thus propose business stand next kin fund decease fund release due process follow transaction totally free risk trouble fund legitimate originate drug money laundry interest let hear please forget include contact detail regard peter
1	good day ei name chajia mariam sani abacha cthe former first lady also wife former late head state federal republic nigeria clate gen esani abacha whose sudden death come th june since death ci thrown astate utterconfusion frustration hopelessness present civilian administration subject physical psychological torture security agent country son mohamm detention arraign federal high court nigeria offence commit ethough release since rd september base certain condition cbut allow move around ehe restrict town also place government watch widow traumatize lose confidence anybodywithin country must hear medium report internet recovery various huge sum money deposit husband different security firm abroad company willingly give secret disclose money confidentlylodg many right blackmail fact total sum discover government far tune emillion

	dollar relent make poor life ei get contact personal research cand desperation decide reach medium ei give information regard soon reply repose great confidence hence approach due security network place day day affair afford visit theembassy decide contact hope betray confidence deposit sum million dollar security firm abroad whose name witheld open communication shall grateful could receive fund account safe keep arrangement know son abba alone cso son deal directly security whole ei seriously consider settle abroad friendly atmosphere like soon fund get account start wish cbut impossible cjust help keep fund account accrue fund please honesty watch word transaction ei require telephone fax number commence communication immediately give detailed picture thing ein case dont accept please let security give information total trust confidence greatly appreciate accept proposal good faith eplease expedite action esincerely chajia mariam abacha
1	dear friend like invest country samuel dlovu liberia son michael dlovu former minister mine industry parent kill liberia war father deposit trunk box contain million dollar global security finance company ghana safe keep death instruct claim look sister self sister accra ghana west africa notify claim fund intend invest money abroad hence contact advise good invest money country assistance offer money vital document cover deposit fund ownership send request please send direct phone fax number information reply thank much anticipate acceptance expect prompt response good regard samuel dlovu prueba el nuevo correo terra seguro aacute pido fiable
1	would like apply medium co operation secure opportunity invest joint business country much knowleagde international business investment substantial capital honorably intend invest countryinto lucrative venture advise execute thesaid venture mutual benefit ableco operation become business partner trustee representativein country create idea money invest probably manage type ofinvestment money transfer custody yourassistance meanwhile indication willingness handle transactionsincerely protect interest upon acceptance thisproposal would furnish full detail information procedure amount involve mutual agreement percentage interest sharehold help secure release deposit investingthe money shall glad reserve respect opportunity sodesire urge give matter immediate attention deserves look forward response mr femia bangura
1	compliment decide contact business transactionhop contact earliestconvenient possible business deal involvingmoney transfer gbp thirty millionbritish pound presently auditor general bank locatedat heart london sincere assistant andco operation determine work deal outif business moment amconstrain issue detail businessuntil response receive familiar aboveinformation please take moment busyschedules read send response thisdeal worth take highly profitable utmost response highly awaited myprivate email address thank time attention warm regard mr linus john delete button history unlimited mail storage click away
1	mr suzan sankohabidjan ivory coastdear one mr suzan sankoh wife late cheif andrewkamara owner two gold field seirra leone later take government husband make director ofthemines rebelforces attack take get yourcontact search internet reputable person hel pme tomanage inherit fund good business son able take sir busy person pray enough timeto consent plight proposal would like confide thati inherited total sum usd twenty three milion unite state dollar late husband chief andrew sankoh killedby rebel soldier sierra leone money move abidjan cote ivoire instructionto transfer consignment london budapest holland collectingcenterand deposit prime security firm late husband special secret arrangement declare consignment family valuabl uesand hope invest later suddedn death could notallow sudden death discover copy mad ein state deposit contain money thoughdeclar family valuable security reason son decide seek refuge ivory coast ar look trust partner overseas whose account accomoda te money coveniently deposit investment managmen behalf also come stay country decide contact rely decide invest money good business venture country base political economic stability myseif son agree theonly partner fund manager regard email message seek assistance respect therefore request arrange possible way money could move country even may require come iv ory coast meet son agree total sum shall give rende ring assistance also earmark expense like travel hotel bill seprate account son whil remain family investment partner trustee please reply email message early convenient time email indicate willingness nice day god bless sincerely suzan sankoh con terra mail obtienes mb de espacio adem de bloqueo antispam internet desde peso al me los mejores productos precios increibles aprovecha nuestra promoci pagos sin intereses con banamex banc omer
1	miss aranima enfandy br unhcr refugee camp br danane br cote ivoire br nbsp br dear one br nbsp br plea assistance br nbsp br pleasure contact investment assistance intend establish country br nbsp br though meet believe one risk confide someone succe sometimes life however mandatory manner compel honour br nbsp br nbsp daughter late markeni enfandy nshili district gikomgoro province republic rwanda father death successful gold cocoa merchant johannesburg south africa ghana barely week father return usual european business trip wednesday th june attack kill unknown assassin together dear mum br nbsp br police could trace killer till today know behind parent brutal kill reason felt fear safety guarantee reason decide relocate help international care abidjan cote ivoire formerly ivory coast save life br nbsp br love much like confidant untimely death dad give information kept document huge amount usd br nbsp br keep family presently money courier company bond

	warehouse call ivsf full information disclose later confirmation acceptance assist br nbsp br decide invest money country anywhere safe enough outside africa security political reason problem abidjan br nbsp br nbsp want stand appoint beneficiary receive consignment behalf since deposit shipment stand good way retrieve deposit br
1	account name mr ejis work theinternational operation department bank inlondon feel quite safe deal thisimportant business though medium internet hasbeen greatly abuse choose reach itbecause still remain fast medium ofcommunication however correspondence isunofficial private treat assuch first like assure thistransaction risk trouble free bothparties want transfer money bankhere london fund transfer cleanorigin owner account foreigner aprogram leader death late primeminister mr rafik hariri huge investment herein united kingdom world amatter fact sum thirty six millionseven hundr fifty nine thousand poundssterlings account london hedeposit family valuable family knowabout deposit routine inspection thati discover dormant domiciliary account bal thirty six million seven hundr fifty ninethousand pound sterling discreetinvestigation also discover accountholder pass away dead leave beneficiaryto account bank approve money toany foreigner former operator ci foreigner certainly sure nobody willcome claim money foreignercan claim money legal claim theaccount holder therefore need cooperation transaction provide necessary information need inorder claim money hop god willnever let future rafik bahaaedine hariri november february marry nazek audi hariri lebanese self madebillionaire business tycoon five timesprime minister lebanon last resignation office october late rafik hariri die february explosive equivalent around kg weredetonat motorcade drove past saint georgehotel lebanese capital informationplease log want transfer money safe foreignaccount abroad know foreigner ican trust know message come youas surprise know butbe sure real genuine business icontact believe let downonce fund go account let hear urgently regard mr ejis
1	interested business please urgently contact follow mail address phone number give detail please endeavour keep business confidentially grateful come forward help proof transaction please reply email addressthanks good regard master nelson keren larry koromba
1	seek financial investment information zimbabwe formerly rhodesia colonial rule gain independence nationalist struggle independence lead mr robert mugabe late joshua nkomo white rule since independence minority white own farmland zimbabwe dominate agriculture aborigine believe still slave land continue work land white master even independence robert mugabe black moses lead person freedom vow revoke ownership white own farm redistribute land landless black move say necessary redress imbalance colonial era struggle freedom affect initiative government amend land acquisition law policy last month give white zimbabwean farmer day quit notice leave lands government family catch web land politics also lead death father see reform reforms government mugabe fed economy shamble profitable safe enough remain zimbabwe decide relocate farm swaziland neighbour country acquire land capital base south africa temporarily live pend time logistics would ready full scale operation swaziland bear mind mr thabo mbeki south african president government play russian roulette zimbabwean land crisis every effort begin earnest move capital base stable country safer capital base million great deal secrecy involve avoid pierce eye smell nose government zimbabwe south africa therefore contact use magnificent business technique transfer financial treasure south africa money presently deposit confidentially security company south africa map gross sum due commission expect honest assistance execute successful transfer money south africa family investment please email immediately interested discus hold correspondence strict confidence thanks god bless truly evan smith
1	dear lord name person south africa marry mr greoge sankoh work south african embassy ivory coast nine year die year marry eleven year without child die brief illness last four day death bear christian since death decide marry get child outside matrimonial home bible late husband alive deposit sum million five million five hunderd thousand dollar trunk box one security finance company cote ivoire presently money still security finance company recently doctor tell would notlast next two year due cancer problem though disturbs stroke know condition decide donate fund church good still christian individual utilize money way go instruct want church use fund church orphanages widow propagate word god ensure house god maintain bible make understand bless hand giveth take decision child inherit money husband relative christian want husband hard earn money misuse unbeliever want situation money use ungodly manner hence reason take bold decision afraid death hence know go know go bossom lord exodus say lord fight case shall hold peace need telephone communication regard health presence husband relatives around always want know development god thing possible soon receive reply shall give contact bank ivory coast also issue letter authority empower original beneficiary fund want church always pray lord shephard happiness live life worthy christian whoever want serve lord must serve spirit truth please always prayerful life delay reply give room search church christian individual purpose please assure act accordingly state herein hop hear soon receive mail remain bless name lord christ sister philomina philomina burtley
1	help secure oil drilling contract year ago ofour interest contract invoice mentionedamount contract company receive actualcontract sum go back country leave behind thisover inflate sum colleague claim time come process move fund forinvestment since still work withthe global security

	finanace ltd need help thati use company process release fund reciv payment execute contract please let know company also let know youcan able receive money behalf invest yourcompany good profitable business country withoutexpos commence process transfer need send immediately full name company name telephone fax numbersyour share assistance take take care expense incur cause oftransfer please write back know capable enough executethis project please transaction require utmostconfidentiality alternative private email address forward cooperation faithfully mark luke
1	compliment day sorry embarrassmentmy letter might cause dr frank bello theexecutive director finance nigeria nationalpetroleum corporation nnpc position strategicofficials head department havein possession since sum dollar sixty million united state dollar amassedthrough invoice foreign contract fullyexecut commission different foreigncontractors duly pay full contract amount follow peaceful life usher currentdemocratic dispensation nigeria colleague decide transfer sum million foreign account operate foreignerfor onward transfer account request yourassistance transfer safeguard sum ofus dollar mutual benefit assist transfer safeguard thisfund entitle total sum belongs colleague gofor reimbursement expense make party imperative note transaction riskfree colleague relevant ministry haveperfect modality successful transferof fund however see absolute confidentiality ourwatchword throughout course transactionfor civil servant want safeguardour job receive pension since haveput lot year active service ungrateful urgently furnishme full name want appear thenecessary document address private telephone faxand mobile number enable conclude allmodalities associate transfer fundsfor onward transmission nominate bankaccount central bank nigeria hasauthoriz guaranty credit bank nigeria tomake payment contractor beinclud among contractor mycolleagues look forward hear soonest stopfurther contact regard dr frank bello email enviado utilizando servi megamail
1	please kindly call mr nosa though complete name give full name later hide benin republic country west african large sum money investment money million dollar move freely reason explain later need help urgently safe keep invest money country one three email contact give cyber cafe operator request foreign contact tell name country origin though tellher need contact foreigner urgent confidential nature thisbusiness advise keep everything secret interested business kindly reply immediately email address send verifiable detail
1	thanks anticipate co operation please include telephone number fax number reply good regard gideon arap moi
1	know surprise read please consider arequest family dire need assistance first must introducemyself mr tsepo vusi angola first son brigadier vusi jones presently resident south africa get contact address business directory johannesburgchamber commerce industry behalf widow mother mrs elizabethvusi desid solicit assistance transfer sum million twenty one million five hundred thousand united state dollar inheritedfrom late father personal company account father death brigadier charge arm andammunation procurement angola army specificallydrew attention say sum money deposit safe box ofa private security company johannesburg south africa treasure box fully document name fact father say quote belov son wish draw yourattention million deposit box contain money securitycompany johannesburg south africa war dedicatedand officer government functionary bussy help withgovernment fund property send foreign country dueto former special advisor president assignedby president eduardo santos purchase arm south africa see thisas golden opportunity divert money divide get total sumof million case absence earth result deathonly solicit fund investment purpose understand live future familydepens money grateful assist nowliv south africa political asylum seeker financial law andregulation republic south africa permit financial right suchhuge sum money view invest fund south africa hencei prepare offer total fund set asidefor local international expense family finally modalities howthe transfer bone convey wehave establish trust confidence please treat thismatter urgent good regard tsepo vusi
1	greeting dear sir greeting compliement season due consideration trust come profitable businesstransaction might interest name zedex calvaho thefamous calvaho family calvaho family polygamous family thrichest family sao tome principe father late dr tinjankabacalvaho die nd march living million dollar andpounds foreign bank include large number estate companieswhich already claim child father first with ecurrently parading sole inheritor father sestates fund caused series problem familyand family late father first wife till date problemhas able resolve even court however able trace father unclaim money tun eof million lie ubs investment bank london conculdedevery arrangement claim fund child father sfirst wife bounce fund inheritance addition since thedeath father side calvaho family serioussevere financial distress first family confiscate allthat father labour interest entirefamilies therefore wish present bank forclaim fund behalf part benefit ourlate father fund fund release nominatedbank account without delay offer fundsfor kind assistance incase offer please thisfunds hope livelihood otherwise turn abegger oneday finally appreciate send directtelephone number discussion deal detail sincerely zedex calvaho
1	get contact internet referal link comfort mensa daughter late mr charles kofi mensa late father charles mensa limited liability cocoa gold merchant liberia umtimely death eafter trip ghana cto negotiate cocoa gold business want invest ghana week come back ghana attack mother unknown

	assassin mother die instantly father die five day private hospital faithful afternoon know father go leave lose mother die know go die father may soul rest perfect peace disclose deposit sum dollar twenty one million eight hundred thousand dollar security company ghana money meant cocoa gold company want establish ghana hand relevant document document deposit box instruct seek reliable trust worthy partner non speculativelife time investment abroad succeed locate security company ghana consequently solicit assistance help get box security company possible come ghana transfer fund account overseas invest meaningful flucrative business country hope source livelihood ei also like know percentage take assistance successful completion transaction soon hear forward vital document send company claim forward tele ffax number word soon hear also forward private telephone number reach mei opinion treat letter strict confidentiality await response regardsms comfort mensa
1	wife ex nigerian president head state late gen sanni abac hawho die th june sometime death heentrust sum fifty million united state dollar wh ichwe deposit organization disclose later thi smoney mean campaign self succession bid unfortunatel yhe die actualization aspiration amount entrust late husband due trustand confidence wife agreement abachas family write solicit assistance transfer sum mto subsequent disbursement solicitingfor assistance present nigerian government freezingany bank account traceable late ex head state family rela tives assistance agree compensate tot alsum major part share shall usedfor investment overseas note transaction risk free map strate giesfor successful transaction organization disclose late please endeavor keep transaction absolutely secret transaction expect conclude within work day yo uare interested would want contact immediately private co nfidentialemail confidential email address need name tel fax firstthree page internationalpasport address know deal thank anticipate kind understanding urgent positive re ponse iwiil email picture money get reply pleasehelp good regard dr mrs mariam abacha abachaprivate
1	change ownership certificate name powerof attorney soon collect insurancepolicy cover send tothem immediate deliver box youraddress charles taylor
1	good day let first treat get email address look forsomeone go partnership order achieve aim endof day leave extra dollar account please note scam honest transaction willbe transact two reputable personality mr vincent soglo chief credit officer international trust housebenin account open firm since nobodyhas operate account go oldfiles record discover remit money outurgently forfeit nothing vault account col ibrahim sankoh credit balance ofus dollar beneficiary col ibrahim sankoh die leavingno possible record trace heirs obvious reason decide inherit balance follow legitimaterelease procedure willing assist please hurry provide yourfull name residence address occupation date birth contact phonenumbers communication purpose unanimously conclude release total sum participation please get back detailed discussion truly mr vincent soglo
1	hon dr adeniji olu minister foreign affair ministry office mo nitors control affair bank financial institution nigeria concerned foreign contract payment final signat ory transfer remittance huge fund move within bank local international level line foreign contract settle ment list fund could transfer minated account account identify either ghost ccounts unclaim deposit invoice sum etc name amon person expect fund transferred account note wish deal regard unpaid fund file hope data correct un tamper ed unless reconfirms duty recommend transfer surplus fund federal government treasury reserve account unclaim deposit opportunity write base instruction receive day ago senate committee contract payment foreign debt submit list payment report expenditure audit report revenue among several others decide remit contract sum follow idea deal agreement go thi legally condition sum usd pay account provide confirm transfer sum account elegraphic transfer confirmable working day deal must keep secret forever correspondence strictly email telephone security purpose third party problem associate fund release cause agent representative agree condition advise immediatelyand transfer commence without delay proceed fix name payment schedule instantly meet three day mandate hope dont reject offer fund transfer wait reply soon hon dr adeniji olu foreign affair minister
1	hellothis letter may come surprise due fact yet meet say intention cause pain decide contact medium read want feel sorry believe everyone die someday name razaq samiu merchant dubai diagnose prostate esophageal cancer discover late due laxity care health defile form medicine right month live accord medical expert particularly live life well never really care anyone even business though rich never generous always hostile person focus business thing care regret know life want make money world believe god give second chance come world would live life different way live know time near will give property asset immediate extend family member well close friend school uae decide give alms charity organization want one last good deed earth far distribute money charity organization england ireland health deteriorate badly self ask member family close one account donate money charity organization bulgaria refuse kept money hence trust anymore seem contend leave last money huge cash deposit bank england uk want help collect deposit dispatch charity organization let know razaq samiu make generous donation write

	laptop computer hospital bed england wait time come interested help give information like amount deposit bank contact bank contcat also send picture self laptop hospital note take fund give charity organization pray god use support assist good heart god
1	amount deposit finance company value need help clearance transfer country want relocate good life talk send mail phone reach want relocate area decide confide detail fund need help clearance transfer want relocate living investment kind help require trust confidence quality need investment fund good investment know able take good care younger brother help control fund clearance account meanwhile tell brother also inform tell come fund need help clearance transfer account investment ask trust without even know good tell brother child god god dissapoint direct contact immediately see profile firstly like tell understand comprehend point clearly name miss quien jelo benin republic young brother hotel call hotel colombo arrive two day big war country call ivory coastr lead death father ad mother living young brother alive sierra leone bo live country call abidjan cote ivoire benin republic enter result war country lead death father mother live david alone earth benin republic enter refugee though refugee camp access internet talk come recently stay hotel please need assitance help writt death father use name deposit money next kin finance company benin republic total amount deposit value million dollar stand beneficiary lay claim allow refugee operate account bank finance company contact come fund clear late father exporter gold diamond european country death need help get total money transfer account investment change beneficiary name favour person authorise empower clearance need follow enable change beneficiary favour standind bonafide empower finance company contact transfer sign power attorney empower release transfer instruct authorised need follow full name address telephone mobile receive telephone call assure readiness willingness help clear claim money assure also help invest money hope live go betray money arrive account submit detail change beneficiary favour enable finance company fund transfer authorise
1	dear friendplease accept apology contact way due urgency request get contact enquiry place chamber commerce trade industry partner need worthy business partner abroad handle investment specify fund lie fallow sometime accept work would foreign patner recieve say fund behalf due sensitive position place work also hold investment travel meet request intimate blueprint recieve mail note please forward reply mail box state security reason make sure include private telephone number easy communication hop hear soon regard mohamm ca dd mon jun webmail postino imp http
1	hello friend good day compliment write letter confidence believe wish god help family god almighty bless reward abundantly youwould never regret family true christian worship god truthfully get contact search assistance female student university burkina faso ouaga yrs old like person care love home orient love havea long term relationship know would like build solid foundation time come able help transaction well father die early three month ago leave junior brother behind hewas king town citizen title sixteen year beforehis death princess person take care wealth junior brother still young mother literate enough know entire father wealth leave sum usd seven million three hundred fiftythousand dollar security company money annually pay late father account shell petroleum development company spdc chevron oil company operate locality compensation youth community development jurisdiction know invest money somewhere abroad father kindr take belongs father family planning without consentbecause female stated culture town urgently need humble assistance move money security company bank account felt happy see contact strongly believe grace god help invest money wisely ready pay total amount help transaction another interest annual income handle transaction strongly absolute control handle project sincerely also willing assist lift fund kindly reach please note transaction risk free hope commence transaction quick possible send picture soon hear recognition sincerely
1	please reply private email addressfrom mr biney saac private email awilsonjohn fbiney ecom attn athe manage director mr biney isaac charge cargo section rochasab security banglamung thailand due respect regard decide contact business transaction beneficial end transaction investigation audit security company department come across consignment belonging decease person die plane crash security company without claim consignment custody either form family relation discovery development although personally keep information secret within partnerto enable whole plan ides profitable successful time execution say amount five million six hundred thousand united state dollar may interest know get impressive information good friend work chamber commerce thailand emean whole arrangement put claim fund bonfire next kin decease relay soon indicate interest willingness assist also benefit yourselfto great deal fact could do deal alone position country civil servant allow operate foreign account would eventually raise eyebrow side time transfer work company actual reason require second party fellow forward claim next kin affidavit trust oath security company also present foreign account need money transfer request may due verification clarification designation back account fail inform transaction risk free smooth conclusion transaction entitle total sum gratification set aside take care expense may arise fine transfer also telephone bill partner please advised keep top secret still service intend retire service conclude deal write application rinse company join business require come country subsequent share find accord percentage previously indicate investment either country country advice invest

	necessary information send heard suggest get back soon possible state wish deal cmailto awilsonjohnbeney yahoo ecom eyours faithfully mr biney isaac
1	audit accounting section african devlopment bank ouagadougou burkina faso west africa attention please mr mose maliki director charge audit account ection african development bank ouagadougou burkina faso wes africa due respect regard decide contact busine ss transaction beneficial end transaction investigation audit bank department come across huge sum money belong decease person die plane crash fund dormant account th bank without claim fund custody either fa mily relation discovery development say amount dollar may interest know get impressive information throu gh internet directory meanwhile whole arrangement put cla im fund bonafide next kin decease get require approval transfer money foreign account put place directive need information relay soon indicate interest willingness assist also benefit self great business opportunity fact could do deal alone position cou ntry civil servant banker allow operate fore ign account would eventually raise eye brow side th time transfer work bank actual reason require second party fellow forward claim next kin affidavit trust oath bank also present foreign account need money transfer request may ue verification clarification correspondent branch ba nk whole money remit designation bank account fail inform transaction risk free smooth conclusion transaction entitle total sum gratification set aside take care expense may arise time transfer also te lephone internet bill coleagues please adviced keep top secret still se rvice intend retire service conclude deal wit monitor whole situation bank unti confirm money account ask come country subsequent share fund accord percentage pr eviously indicate investment either country country advice invest necessary vital nformation send hear look forward receive phone fax number easy communica tion faithfully mr mose maliki mile de amigos con tus mismas aficiones
1	quien pueda interesar soy soldado americano estoy desempen ando servicios en los militares del primero divisi armada en iraq como usted sabe cerca nos est atacando insurrectos diarios bombas de coche manejamos mover los fondos que pertenec edan la familia de saddam hussein la cantidad total lares de million en efectivo sobre todo cuentas de lar deseamos mover este dinero usted de modo que ust pueda invertirlo para nosotros guarde nuestra parte para la actividades bancarias tomaremos el mi socio yo usted toma el otro ningunas secuencias unidas justo ay fadenos moverlo de iraq iraq una zona de la guerra planeamos en usar el mensajero diplom tico enviar el dinero hacia fuera en una caja de plata grande usar inmunidad diplom tica si ust soy interesado le enviar los detalles completos mi trabajo est al hallazgo buen socio que podemos confiar en que no asistir bfpuedo confiarle en cuando ust recibe esta letra amablemente env edeme significar del mail su inter incluyendo su tel fono confidencial fameros de fax para comunicaci pida tambi sus detalles del contacto este negocio riesgo libremente la caja se puede enviar hacia fuera en hr respetuosamente
1	dear name mr anna william die woman decide donate church year old diagnosed cancer year ago immediately death husband leave everything work touched god donate inherit late husband good work god rather allow relative use husband hard earn fund ungodly please pray good lord forgive sin ask god forgive believe merciful god go operation less four hour decide towill donate sum nine million five hundred thousand dollar good work lord also help motherless less privilege also assistance widow accord james moment take telephone call right due fact relatives around health status adjust lawyer aware change lawyer arrange transfer fund security company wish good may good lord bless abundantly
1	letter mr dennisdear partner name mr dennis stevenson chairman board director bank scotland united kingdom write seek help assistance wish make transfer money involve huge amount money worth fifteen million pound sterlings pound sterling propose make transfer bank account choice thus need help support propose offer total amount share transfer successfully conclude family expense make transfer process kindly reply state interest shall give detail necessary proceedure make transfer anxiously await response reply soonest thanks godbless thanks god bless mr dennis stevenson
1	hello name david elli nationality unitedkingdom diagnose esophageal cancer defile allforms medical treatment andright month live accord medical expert notparticularlyliv life well never really care foranyone even business though amvery rich never generous always hostileto person focus business wasthe thing care regret thisas know life justwant make money world ibelieve god give second chance come tothis world would live life different way fromhow live god call will givenmost property asset immediate andextend familymembers well closefriends want god merciful accept mysoul decide give alms charityorganizations want one lastgood deed earth far distribute money charityorganizations somalia malaysia health deteriorate badly cannotdo anymore asked member myfamily close one account distribute themoney charity organization inbulgaria pakistan refuse kept moneyto hence trust anymore asthey seem contend leftfor last money one know hugecash deposit twenty eight million usd havewith finance security company abroad wantyou help collect deposit despatch tocharity organization set aside time god david elli

1	since get information death furtherinvestigation find die without make attempt trace next kin fruitless therefore make investigation discover thatmr raymond beck declare kin relation inallhis official document include bank deposit paperworkin bank sum still sittingin bank interest roll theprincipal sum end year one ever comeforward claim accord law united arab emirate expiration seven year money willrevert ownership government ifnobody apply claim fund consequently proposal like foreigner stand next kin mr raymond beck fruitsof old man labor get hand ofsome corrupt government official simple like provide immediatelyyour full name address attorney willprepare necessary document affidavit thatwill put place next kin shall employ service attorney draftingand notarization obtain necessarydocuments letter probate administration yourfavor transfer bank account inany part ofthe world provide facilitatethe transfer money beneficiaryext kin money pay accountfor share ratio foryou risk paperwork thistransaction do attorney positionas branch manager guarantee successful executionof transaction interested please replyimmediately via private email address uponyour response shall provide moredetails relevant document help youunderstand transaction please send confidential telephone faxnumbers easy communication please observe utmostconfidentiality rest assure transaction would profitable ofus shall require assistance toinvest share country await urgent reply via confidential email haitham sharaf dfe abb ecf abe
